# *EF1α* and *αTUB* Are Stable Reference Gene Pairs for RT-qPCR-Based Gene Expression Studies in *Salix suchowensis* Under Nitrogen Treatment Conditions

**DOI:** 10.3390/plants14193101

**Published:** 2025-10-08

**Authors:** Lei Huang, Yuyi Zhang, Fei Gao, Yu Fu, Jing Sun, Jie Zhou, Jun Tao, Xudong He, Nan Guo

**Affiliations:** 1 College of Horticulture and Landscape Architecture, Yangzhou University, Yangzhou 225009, China; hhlei56@163.com (L.H.); 18796605690@163.com (F.G.); jingsun@yzu.edu.cn (J.S.); jtaoyzu@163.com (J.T.); 2 Jiangsu Key Laboratory for Conservation and Utilization of Plant Resources, Institute of Botany, Jiangsu Province and Chinese Academy of Sciences (Nanjing Botanical Garden Memorial Sun Yat-Sen), Nanjing 210014, China; zhangyuyi@jib.ac.cn; 3 National Willow Engineering Technology Research Center, Jiangsu Academy of Forestry, Nanjing 211153, China; zjwin718@126.com

**Keywords:** *Salix suchowensis*, nitrogen, reference gene, RT-qPCR

## Abstract

*Salix suchowensis* is an ideal model organism for investigating nitrogen (N) transport mechanisms due to its low N-input requirements. Accurate quantification of gene expression is essential for elucidating these processes, with quantitative real-time PCR (RT-qPCR) being the preferred method. However, the identification of stable reference genes for normalization in *Salix suchowensis* under varying N conditions remains unresolved. In this study, thirteen commonly employed candidate reference genes were evaluated across root, stem, and leaf tissues, under four N treatments (NH_4_NO_3_, NH_4_^+^, NO_3_^−^, and N deficiency). Five genes (*UBQ1*, *UBQ3*, *18S*, *H2A2*, and *H2B2*) were excluded due to poor amplification efficiency or irregular melting curves. The remaining eight genes were further assessed for expression stability using the geNorm, NormFinder, and BestKeeper algorithms. Integrated ranking via RefFinder identified *EF1α*, *EFβ*, and *αTUB* as the most stable reference genes. GeNorm analysis suggested that two reference genes were sufficient for reliable normalization. Validation using the N-responsive gene *SsAMT1* and *SsNRT2* confirmed the stability of *EF1α*, *EFβ*, and *αTUB* as suitable reference genes. Based on comprehensive stability assessments and experimental validation, we recommended *EF1α* + *αTUB* as optimal reference gene pairs for RT-qPCR normalization under varying N conditions. Furthermore, the consistent expression of *EF1α* and *αTUB* across nine willow genotypes highlighted their broader applicability within *Salix* species. This study provides valuable methodological guidance for advancing molecular research on N transport in woody perennial plants.

## 1. Introduction

Nitrogen (N) is a macronutrient for plant growth and development [1]. In soils, N exists in both inorganic and organic forms, with inorganic ammonium (NH_4_^+^) and nitrate (NO_3_^−^) representing the primary inorganic N sources taken up by plants. Typically, NO_3_^−^ is dominate in well-aerated soils, whereas NH_4_^+^ tends to accumulate in acidic or anaerobic environments where nitrification is inhibited [2,3,4]. The preference for NH_4_^+^ or NO_3_^−^ as a primary N source is fundamentally dependent on plant genotype, which reflects the species’ evolutionary adaptation to its native soil environment. For instance, conifers from boreal forests exhibited a marked preference for NH_4_^+^, having evolved on acidic, NH_4_^+^-rich soils where nitrification is low [5]. Meanwhile, species like *Populus* spp. were often assumed to prefer NO_3_^−^ due to their association with floodplains and high nitrate reductase activity [6]. Moreover, acid-tolerant species such as *Oxalis acetosella*, adapted to high-acid environments, grew equally well with NH_4_^+^ alone. In contrast, *Urtica dioica*, a species from less acidic soils, strongly favored the presence of NO_3_^−^ [7]. Different N forms distinctly influence plant physiological responses, especially affecting root system architecture [8]. As the principal site for N uptake, roots exhibit high morphological plasticity in response to environmental cues [9]. Studies have demonstrated that NO_3_^−^ supply stimulates the elongation of primary and secondary lateral roots, whereas NH_4_^+^ availability enhances the density of secondary and tertiary lateral roots, resulting in shorter but densely branched root systems [10,11]. A high concentration of NH_4_^+^ negatively impacts leaf development by inhibiting cell expansion, impairing osmotic adjustment, and disrupting hormonal signaling. Ammonium-preferring plants usually exhibit smaller leaf area and lower leaf biomass compared to nitrate-preferring plants, ultimately resulting in reduced carbon accumulation [12]. In contrast, NO_3_^−^, apart from a portion stored in vacuoles, needs to be reduced to NH_4_^+^ before being assimilated into amino acids for protein synthesis, a process that is an energetically demanding [13,14]. Despite this energy requirement, NO_3_^−^ as a sole N source does not exert toxic effects on plant growth, whereas NH_4_^+^ can be toxic and often results in growth inhibition [15,16].

*Salix suchowensis* is a compact shrub willow predominantly found in temperate regions of East and Central China [17,18]. Typically reaching heights of up to 3 m, it forms dense stands that contribute significantly to ecosystem stability. Remarkably, *Salix suchowensis* achieves sexual maturity within one year, allowing multiple harvests within short timeframes [19]. This trait makes it particularly suitable for short-rotation coppice (SRC) systems, in which cyclical harvesting occurs at intervals of 2–3 years [20]. The species’ natural ability to redistribute nutrients during its perennial growth cycle supported its classification as a low-N-input crop [21], enhancing its values for urban landscaping, as well as for bioenergy and industrial applications [19]. Physiological studies have further revealed that *Salix suchowensis* possessed exceptional N-use traits. It was noted that even minimal N levels (10 μM NH_4_^+^) were sufficient to stimulate the growth. A series of ^15^N-labeling assays revealed that within 1 min of NH_4_^+^ exposure, the process of uptake, assimilation into amino-N, and transport to aerial tissues were completed. This rapid N uptake and transport abilities underlined the species’ adaptation to environments with extremely low N availability [22], a trait not reported in other willow species. Despite these insights, the molecular mechanisms underlying N uptake and source-to-sink transport in *Salix* remained largely unknown. Importantly, the completion of the *Salix suchowensis* genome provides a valuable resource for exploring the genetic mechanisms of N transport processes in perennial woody species [23]. Investigating the spatial and temporal expression patterns of candidate genes will be essential for elucidating their functional roles.

Quantitative real-time PCR (RT-qPCR) has been widely applied for analysis of gene expression patterns, owing to its high accuracy, reproducibility, sensitivity, and high-throughput capability [24,25]. To ensure the accuracy and reliability of RT-qPCR results and control for variations between and within samples, standardization using one or more internal reference genes was essential [26]. These reference genes serve as internal controls to normalize gene expression data and minimize experimental errors [27]. However, no single reference gene exhibits universally stable expression among different plant species, tissues, and experimental conditions [28]. For example, in peach, *TEF2* (*Translation elongation factor 2*), *UBQ10* (*Ubiquitin protein 10*), and *RPII* (*RNA polymerase II*) were the most stable reference genes [29], whereas in rice, *UBQ5* and *eEF-1α* (*Elongation factor-1α*) were suitable [30]. In pepper, *UBI-3* (*Ubiquitin-conjugating protein*), *β-TUB* (*Beta tubulin*), and *GAPDH* (*Glyceraldehyde-3-phosphate dehydrogenase*) were identified as the most stable reference genes [31]. In addition, even within the same species, different tissues or treatment conditions can influence the stability of internal reference genes. In *Salix* species, *ACT* (*Actin*) and *DnaJ49* (*Chaperone protein DnaJ49*) were found to be the most stably expressed reference genes in male and female flowers of *Salix suchowensis* at different stages of development [32]. In *Salix matsudana*, *α-TUB2* and *DnaJ49* were stably expressed across different tissues under various stress conditions [33], while in *Salix viminalis* leaves, *TIP41* (*Type 2A phosphatase Activator*) exhibited the highest stability [34]. In *Salix psammophila*, the optimal reference genes selected for gene expression analysis were *UBC* (*Ubiquitin-conjugating enzyme E2*) and *LTA4H* (*Leukotriene A-4 hydrolase homolog*) for heat or drought treatment, *HIS* (*Histone superfamily protein H3*) and *ARF2* (*ADP-ribosylation factor 2*) for cold treatment, *OTU* (*OTU-like cysteine protease family protein*) and *Actin 7* for salt treatment [35]. Although reference gene studies existed in other willows, suitable reference genes for RT-qPCR normalization under variable N conditions and across diverse tissues in willow species have not been identified. Given the genomic resources of *Salix suchowensis* and its unique N physiology, identifying and validating stable reference genes is essential for supporting accurate gene expression studies in this system.

To address this gap, the present study aimed to identify stable reference genes for accurate gene expression analysis in high N-use efficiency *Salix suchowensis* exposed to different N forms across root, stem, and leaf tissues. Thirteen candidate reference genes (*EF1α*, *EFβ*, *αTUB*, *βTUB*, *GAPDH*, *18s*, *Actin1*, *Actin2*, *H2A1*, *H2A2*, *UBQ1*, *UBQ3*, and *H2B2*) were selected for evaluation. The stability of these candidate genes was systematically assessed using geNorm, NormFinder and BestKeeper. Comprehensive analysis identified the *αTUB/EF1α* or *αTUB/EFβ* combinations as the most suitable reference gene pairs for RT-qPCR normalization in *Salix suchowensis* tissues under varying N treatments. Furthermore, the stability and suitability of the selected reference genes were validated under long-term N treatments and across diverse *Salix* genotypes, with N-responsive target genes further confirming their reliability. The findings provide a crucial foundation for future molecular studies on N uptake and transport mechanisms in *Salix suchowensis* and related woody plants.

## 2. Results

### 2.1. Verification of Primer Specificity and Amplification Efficiency

Thirteen commonly used candidate reference genes (*EF1α*, *EFβ*, *αTUB*, *βTUB*, *GAPDH*, *18s*, *Actin1*, *Actin2*, *H2A1*, *H2A2*, *UBQ1*, *UBQ3*, and *H2B2*) were initially selected for analysis in *Salix suchowensis* (Table 1, File S1). Primer amplification efficiency and specificity were assessed using PCR and RT-qPCR. The amplification products of these candidates were 100–200 bp in length (Figure S1A). Among these, *UBQ1*, *UBQ3*, and *H2B2* were minimally detectable and the RT-qPCR melting curve revealed that *UBQ1* and *H2B2* did not yield a single melting peak (Figures S1 and S2). Consequently, *UBQ1*, *UBQ3*, and *H2B2* were excluded from further analysis. Analysis of PCR gel electrophoresis, RT-qPCR cycle threshold (Ct) values, and melting curves indicated that the remaining ten candidate reference genes produced distinct single bands, Ct values below 30, and single-peaked melting curves (Figures S1 and S2). Standard curves were further constructed for other ten candidates, showing that the amplification efficiencies of *18S* and *H2A2* were 237% and 155%, respectively, exceeding acceptable ranges (Figure S3). The remaining eight primer pairs demonstrated amplification efficiencies within the acceptable range of 90–110% and exhibited linear regression correlation coefficients (R^2^) greater than 0.99 (Figure S3). Based on their amplification efficiency and product specificity, these eight candidate reference genes were selected for further stability evaluation.

### 2.2. Expression Profiles of Reference Genes in Roots, Stems, and Leaves Under N Treatments

**Figure 1:**
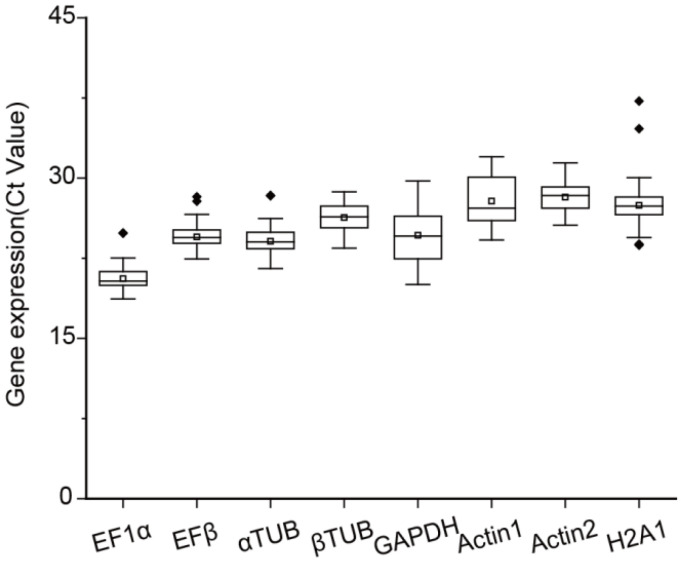
Expression profile of the eight candidate reference genes in root, stem, and leaf tissues of *Salix suchowensis* seedlings under different N treatments for 24 h. The boxplot presented the concentrated range of the cycle threshold (Ct) values, with the horizontal line inside representing the median, the white box indicating the mean, and the dots (◆) denoting outliers.

In gene expression analysis, the Ct value is a critical parameter reflecting the expression level of a gene, with lower Ct values indicating higher gene expression. Ideally, the Ct values of a reference gene should fall within the range of 15–30. In this study, Ct values of eight candidate reference genes were measured across various experimental conditions, including different tissues (roots, stems, and leaves) and N treatments (NH_4_NO_3_, NH_4_^+^, NO_3_*^−^*, and −N) (Figure 1). Among the candidate genes, *EF1α* exhibited the highest average expression level, with an average Ct value of 20.59, while *Actin2* showed the lowest average expression level, with an average Ct value of 28.21. The Ct values for *βTUB* ranged from 23.44 to 28.71, displaying the least variation in the expression levels among the eight candidates (Figure 1). In contrast, *H2A1* exhibited the most variability, with Ct values being 23.72–37.21 (Figure 1). These findings highlighted significant differences in the expression levels of these candidate reference genes across tissues and treatments.

### 2.3. Expression Stability Analysis of the Selected Eight Candidate Reference Genes

**Figure 2:**
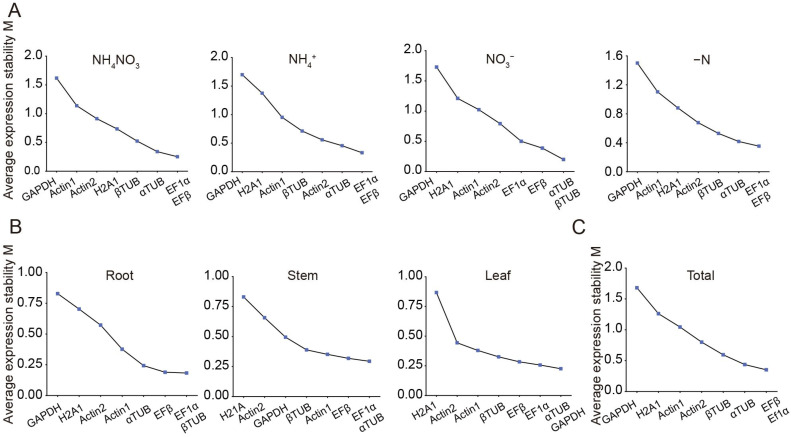
The average expression stability values (M) of the eight candidate reference genes based on geNorm algorithm. M values of candidate reference genes across different nitrogen treatments (**A**), organs (**B**), and the entire sample set (**C**). Genes with lower M values exhibited higher stability. The least stable genes were shown on the **left**, while the most stable ones were displayed on the **right**.

**Figure 3:**
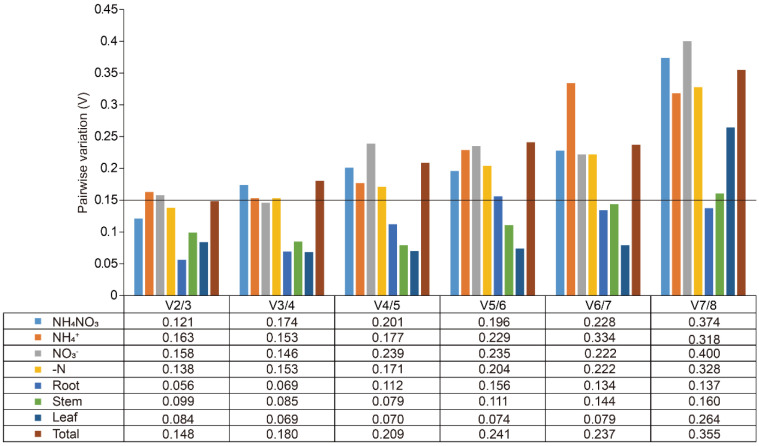
Pairwise variation (V) of the eight candidate reference genes in *Salix suchowensis*. V was calculated using geNorm to identify the optimal number of reference genes required for accurate normalization under different treatments or various tissues, using a threshold of 0.15.

The expression stability of eight candidate reference genes was assessed using geNorm, NormFinder, and BestKeeper tools. GeNorm analysis was conducted to calculate the M values of these genes, where the M value serves as an indicator of expression stability. Genes with M values greater than 1.5 were considered unsuitable as reference genes due to their lower stability, whereas those with M values below 1.5 were deemed appropriate for use [26]. Under N treatments, all candidates, except *GAPDH*, had M values below 1.5, signifying their suitability as reference genes (Figure 2A, Table 2). Among these, *EF1α*/*EFβ* and *αTUB*/*βTUB* exhibited the smallest M values, making them the most stable reference genes under N treatments (Figure 2A, Table 2). Additionally, the M values of all reference genes across different organs (roots, stems, and leaves) were below 1.5, highlighting their relatively stable expression patterns (Figure 2B, Table 2). Specifically, *EF1α* and *αTUB* were the most stable reference genes in roots, stems, and leaves, respectively (Figure 2B, Table 2). Under all experimental conditions, *EFβ* and *EF1α* had an M value of 0.35, indicating they were the most stable internal reference genes overall (Figure 2C, Table 2). GeNorm was further used to calculate the paired variation (V) values, which determine the optimal number of reference genes required for normalization (Figure 3). The paired variation threshold was set at 0.15, with Vn/n + 1 < 0.15 indicating the optimal number of reference genes. Across all experimental conditions, the variation value V2/3 was 0.148, which is less than 0.15, suggesting that two reference genes were sufficient for accurate normalization (Figure 3).

The NormFinder algorithm evaluated gene expression stability by employing an analysis of variance to calculate the stability value (S value) [36]. A lower S value indicated greater stability of gene expression. In the experimental samples subjected to various N treatments, *αTUB* emerged as the most stable reference gene under NH_4_NO_3_ and NO_3_*^−^* treatments, whereas *EFβ* exhibited the highest stability under NH_4_^+^ and −N treatment (Table 3). When analyzing different plant organs, *αTUB* was identified as the most stable reference gene in both roots and leaves, while *EF1α* demonstrated the highest stability in stem tissues (Table 3). Overall, *EFβ* proved to be the most consistently stable reference gene, displaying superior stability across both N treatments and different organs (Table 3). This suggests *EFβ*’s suitability as a reliable internal control for normalizing gene expression data in a wide range of experimental conditions.

The Bestkeeper software evaluated the raw Ct values of each candidate reference gene obtained through RT-qPCR and ranked their stability based the standard deviation (SD) and coefficient of variance (CV). A lower SD value indicated higher stability in gene expression. When analyzed across both N treatments and different organs, the stability ranking of the eight candidate reference genes was as follows: *EFβ* > *EF1α* > *αTUB* > *βTUB* > *Actin2* > *H2A1* > *Actin1* > *GAPDH* (Table 4). This ranking highlighted *EFβ* as the most stable reference gene, while *GAPDH* exhibited the lowest stability.

**Figure 4:**
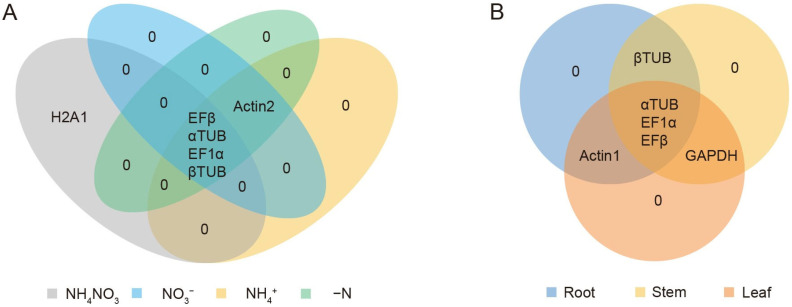
Venn diagram analysis of the top five most stable candidate reference genes for different nitrogen treatment (**A**) or various tissues (**B**). The top five most stable genes were generated by RefFinder: https://blooge.cn/RefFinder/ (accessed on 3 January 2024).

The results obtained from geNorm, NormFinder, and BestKeeper showed some variations in the stability rankings of the candidate reference genes. To address these differences and ensure a more comprehensive and reliable evaluation, RefFinder was employed to integrate and rank the results from these algorithms. This approach provided a more robust analysis of the stability of the eight candidate reference genes. Based on the RefFinder analysis, *αTUB*, *EFβ* and *EF1α* were identified as the most stable reference genes (Table 5). To further explore the overlap of candidate reference genes under different conditions, a Venn diagram was generated to illustrate the shared stable reference genes across both N treatments and various plant organs. The results revealed that *EFβ*, *αTUB*, *EF1α*, and *βTUB* were stable under N treatments, while *αTUB*, *EF1α*, and *EFβ* were consistently stable across different organs (Figure 4).

### 2.4. Validation of the Stability of the Selected Candidate Reference Genes

**Figure 5:**
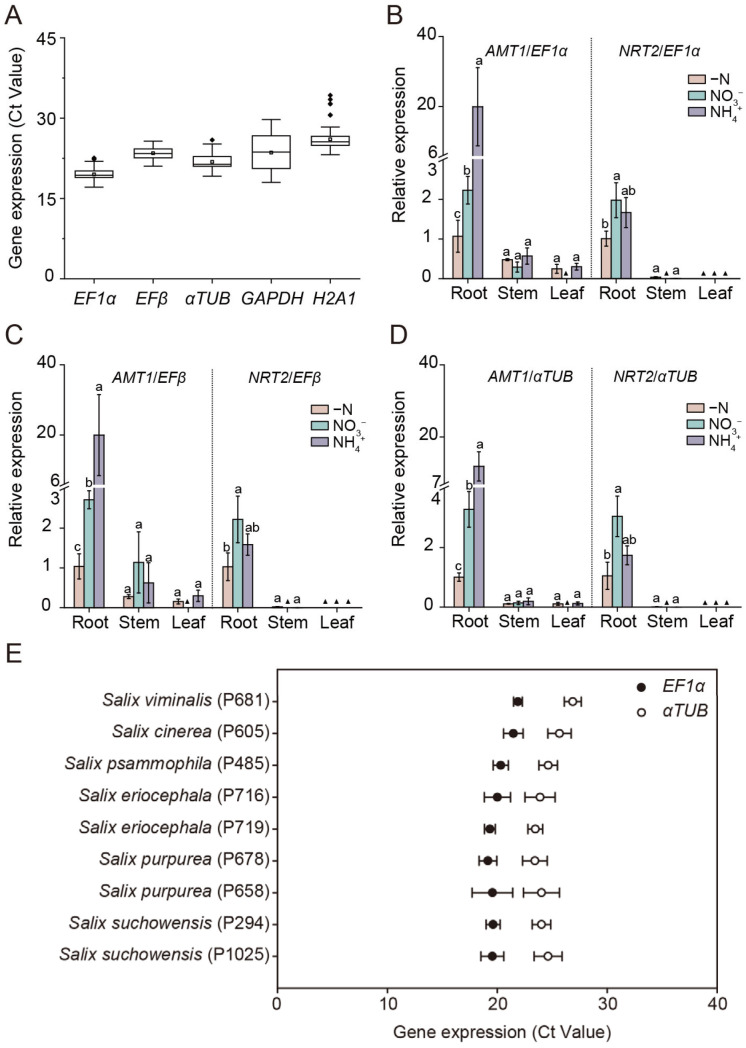
Validation of the stable reference genes under long-term nitrogen regimes, with target genes, and across different *Salix* genotypes. (**A**) Expression profiles of the most stable (*EF1α*, *EFβ*, *αTUB*) and the least stable (*GAPDH*, *H2A1*) reference genes in roots, stems, and leaves of *Salix suchowensis* seedlings under different nitrogen (N) treatments for 5 days. Boxplots show cycle threshold (Ct) distributions, with the horizontal line indicating the median and the white box the mean. (**B**–**D**) Expression of *ammonium transporter 1 (SsAMT1)* and *nitrate transporter 2 (SsNRT2*) in roots, stems, and leaves of *Salix suchowensis* seedlings exposed to different N treatments for 24 h. Relative expression levels were normalized to *EF1α* (**B**), *EFβ* (**C**), and *αTUB* (**D**), and calculated using the 2^−ΔΔCt^ method, with gene expression in roots under −N treatment serving as the control. Different letters indicate significant differences at *p* < 0.05 of the same gene among different N treatments. (▲) Value lower than 0.001. (**E**) Expression profile of *EF1α* and *αTUB* in roots of diverse *Salix* genotypes. Values in panels (**B**–**E**) are means ± SD (*n* = 3–4 biological replicates).

To evaluate the stability of the selected reference genes under prolonged N regimes, we assessed the expression of the most stable (*EF1α*, *EFβ*, *αTUB*) and the least stable (*GAPDH*, *H2A1*) reference genes in the roots, stems, and leaves of *Salix suchowensis* subjected to different N treatments for 5 days. The Ct values for *EF1α*, *EFβ*, and *αTUB* remained within narrow ranges (17.12–22.55, 21.03–25.71, and 19.17–25.93, respectively), indicating stable expression, whereas *GAPDH* and *H2A1* exhibited greater variability, with Ct values ranging from 18.01 to 29.74 and from 23.18 to 34.26, respectively (Figure 5A).

To further verify reference gene performance under N-responsive conditions, we examined the expression patterns of *ammonium transporter 1 (SsAMT1)* and *nitrate transporter 2 (SsNRT2*), two well-established marker genes expressed in root cells that mediated the uptake and transport of NH_4_^+^ and NO_3_*^−^* from the soil [37,38,39,40]. Normalization using *EF1α*, *EFβ*, *αTUB* or their combination yielded consistent expression profiles (Figure 5B–D and Figure S4). Specifically, *SsAMT1* exhibited higher expression levels in roots and was upregulated by NH_4_^+^ treatment, while the transcript abundance of *SsNRT2* was much higher in roots than that in stems or leaves, and its expression was also strongly induced by NO_3_*^−^* supply (Figure 5B–D and Figure S4). The results confirmed that *EF1α*, *EFβ* and *αTUB* were highly stable reference genes for normalizing gene expression in the roots, stems and leaves of *Salix suchowensis* under various N treatments. Considering that *EF1α* and *EFβ* belong to the same gene family, it was recommended to select *EF1α* + *αTUB* or *EFβ* + *αTUB* as the optimal combination of reference genes for normalization in *Salix Suchowensis*.

To extend validation across genetic backgrounds, we further examined the Ct values of *EF1α* and *αTUB* in the roots of nine willow genotypes. Both genes displayed narrow Ct ranges (17.51–22.42 and 22.23–27.69, respectively) with minimal variation among genotypes (Figure 5E), supporting their robustness and broader applicability as reference genes across *Salix* species.

## 3. Discussion

RT-qPCR remains a cornerstone method for gene expression analysis due to its sensitivity, specificity, and reproducibility [41]. However, its accuracy highly depends on the stability of internal reference genes, which serve as critical normalization controls to account for technical variability. Indeed, these genes should exhibit stable expression across diverse tissue types and experimental conditions [42]. However, expression stability is often context-dependent. For example, a gene stable in floral tissues may vary significantly in vegetative organs due to differences in metabolic activity and gene regulation. In *Salix suchowensis*, *ACT* displayed consistent expression in male and female flowers at varying developmental stages but fluctuated across roots, stems, and leaves (Figure 2, Table 4) [32]. The results aligned with the findings that many reference genes commonly used in vegetative tissues are not stably expressed in seeds and pollen [43,44]. Given its potential as a model for studying N transport in woody plants [22], validating suitable reference genes in *Salix suchowensis* under varying N conditions is essential.

Studies across plant species have consistently shown that no single reference gene maintains universal stability under N treatments. For instance, *CACS* (*Clathrin adaptor complex subunit*), *TIP41*, *F-box* protein and *EFα* were most stable in cucumbers [45], while *ELF1B* (*Eukaryotic elongation factor 1β*) and *ACT11* (*Actin*) performed best in soybean [46]. Similarly, *Actin* and *α-Tub* (*α-tubulin*) were most reliable in salt-tolerant plant *Salicornia europaea* [47]. These examples underscore the need for species- and condition-specific validation, as gene stability is influenced by both genetic background and environmental conditions. In this study, *EF1α*, *EFβ*, and *αTUB* were identified as the most stable reference genes in *Salix suchowensis* across tissues and varying N treatment conditions (Figure 4, Table 5). Their stability likely reflected their roles in fundamental cellular processes less influenced by N fluctuations. In contrast, *Actin*, despite its widespread use, exhibited relatively low expression stability under the tested conditions (Figure 2, Table 5), reinforcing the importance of empirical validation over traditional assumptions.

Plant EF1α and EF1β are involved in protein synthesis, during which the nascent polypeptide chain extends by one amino acid residue during one elongation cycle. Therein, EF1α delivered aminoacyl-tRNA to the ribosomal A-site during elongation [48]. It has been reported as one of the most stable internal references under diverse abiotic stress conditions in pearl millet [49], in different tissues of rice [50], and In *Populus euphratica* under cold stress [51]. Similarly, *EFβ* has been shown to maintain stable expression during soybean development [52] and was also identified as a universally reliable reference gene in *Fucus distichus* [53]. *αTUB*, a key regulator of microtubule form and function, enables plants to dynamically sculpt their cytoskeleton for development, stress adaptation, and cellular innovation [54,55]. It has demonstrated high stability in cucumber treated with multiple hormones [56] and across various experimental conditions in *Toona ciliata* [57]. Stability assessments using geNorm, NormFinder, and BestKeeper revealed both overlaps and discrepancies in gene rankings. GeNorm identified *EF1α*, *EFβ*, and *αTUB* as the most stable genes across all conditions (Figure 2). NormFinder supported *αTUB* and *EFβ* as top candidates under N treatments and *EF1α* as the most stable in stems (Table 3). BestKeeper, using a different statistical approach, also ranked *EFβ*, *EF1α*, and *αTUB* among the top candidates (Table 4). These consistent results across multiple algorithms enhance the reliability of the selected reference genes. Notably, geNorm’s paired variation value confirmed that two reference genes are sufficient for reliable normalization, reducing experimental burden without compromising accuracy (Figure 3). Normalizing data with two or more reference genes provides greater reliability compared to using a single gene [36,58]. Importantly, functional validation using *SsAMT1* and *SsNRT2* confirmed that normalization with *EF1α*, *EFβ*, and *αTUB* produced biologically meaningful expression patterns (Figure 5B–D), consistent with the roles of *AMT1* in NH_4_^+^ uptake and *NRT2* in NO_3_^−^ transport [40]. This demonstrated that appropriate reference gene selection not only improves data reliability but also ensures correct interpretation of nutrient-responsive expression profiles. The observed stability of *EF1α* and *αTUB* across nine willow genotypes further suggested their utility in natural populations (Figure 5E). Nevertheless, the present evaluation was restricted to root tissues. Reference gene stability can be influenced by multiple factors, including primer efficiency, experimental treatments, and tissue specificity. Thus, while our results provided valuable preliminary evidence, the utility of *EF1α* and *αTUB* as stable reference genes across diverse willow genotypes requires further validation. Comprehensive assessments under a wider range of conditions and tissues would strengthen their application in future gene expression studies of natural willow populations. In *Salix suchowensis*, *EF1α* and *αTUB* are evidently stable reference gene pairs for RT-qPCR-based gene expression analyses under N treatments.

In conclusion, our findings emphasized that reference gene stability is not universal and that commonly used genes such as *Actin* may not be appropriate in all contexts. Therefore, the selection of reference genes should be guided by empirical validation rather than convention. In this study, the identification of *EF1α*, *EFβ*, and *αTUB* as robust reference genes was valuable for *Salix* research. Their applicability across tissues, N treatments, and genotypes enhanced the reproducibility of RT-qPCR studies and established a methodological foundation for future work on N metabolism and broader physiological processes in woody plants. Further studies evaluating these genes under additional stress conditions would help to establish even broader applicability and enhance the reliability of gene expression normalization in forest genetics research.

## 4. Materials and Methods

### 4.1. Growth of Salix suchowensis and Harvest

Cuttings (1 cm in diameter and 12 cm in length) taken from annual branches of willow species were cultivated hydroponically under natural light in a greenhouse at Yangzhou University, following the method described by Guo et al. [59]. Briefly, uniform cuttings were initially grown in plastic containers with 14 L of water for three weeks, then transferred to 1/4-strength modified Hoagland nutrient solutions for one week. After four weeks, the seedlings of *Salix suchowensis* (ecotype NANJING) were moved to N-free nutrient solutions for one week. Subsequently, they were treated with solutions containing 0.3 mM NH_4_NO_3_, 0.3 mM NH_4_^+^, 0.3 mM NO_3_^−^, or without N for 24 h and 5 days. After the treatment, roots, stems, and leaves of each plant were harvested and immediately frozen in liquid N for RNA extraction. For the validation assay across different willow genotypes, seedlings of nine genotypes of willows were grown for five weeks in Hoagland nutrient solutions. Subsequently, root tissues from each genotype were then harvested for RNA extraction. The solutions were refreshed every three days. Each treatment was performed with four biological replicates.

### 4.2. RNA Extraction, First-Strand cDNA Synthesis, and RT-qPCR Analysis

Total RNA of willow tissues was isolated using the FastPure Universal Plant Total RNA Isolation Kit (Vazyme, Nanjing, China) and reverse transcribed into cDNA using the HiScript II Q RT SuperMix with a gDNA wiper (Vazyme, Nanjing, China). RT-qPCR was conducted with the ChamQ SYBR qPCR Master Mix (Vazyme, Nanjing, China) on a BioRad CFX Connect Real-Time PCR Detection System (Bio-Rad, Hercules, CA, USA). Each reaction was carried out in a 20-μL mixture containing 10 μL of ChamQ SYBR qPCR Master Mix, 0.4 μL of forward primer, 0.4 μL of reverse primer, 1 μL of cDNA, and 8.2 μL of distilled water. Each cDNA sample was analyzed in two technical replicates.

### 4.3. Selection of Candidate Reference Genes and Primer Design

The full-length sequences of thirteen candidate reference genes (*EF1α*, *EFβ*, *αTUB*, *βTUB*, *GAPDH*, *18s*, *Actin1*, *Actin2*, *H2A1*, *H2A2*, *UBQ1*, *UBQ3*, and *H2B2*) were identified using a manual BLAST search: “https://blast.ncbi.nlm.nih.gov/Blast.cgi (accessed on 23 August 2023)” in the *S. suchowensis* genome (Table 1, File S1). Quantitative primers for these genes were designed using the Genscript online tool: “https://www.genscript.com.cn/tools/real-time-pcr-taqman-primer-design-tool (accessed on 7 September 2023)” (Table S1, File S1). The primer design parameters included a GC content of 45–65%, an optimal melting temperature (Tm) of 55–65 °C, primer lengths between 18 and 25 bp, and amplicon lengths ranging from 80 to 200 bp. Primer specificity was confirmed through BLAST analysis against *S. suchowensis* genome. Primers were then synthesized by Tsingke Biotech Co., Ltd. (Nanjing, China), and their amplicon size and specificity were verified by PCR followed by 3% agarose gel electrophoresis and RT-qPCR.

### 4.4. Establishment of Reference Gene Primer Standard Curve

The standard curves were established using a gradient dilution of cDNA (5^0^, 5^−1^, 5^−2^, 5^−3^, 5^−4^, 5^−5^, 5^−6^, and 5^−7^), with the dilution factors plotted on the x-axis and the corresponding Ct values on the y-axis [60]. Slope analysis was conducted using linear regression models. RT-qPCR efficiency (E) was calculated using the formula: E = (10^[−1/slope]^ − 1) × 100 [61]. Internal reference genes with E ranging from 90% to 110% and r^2^ > 0.99 were selected.

### 4.5. Statistical Analysis

The stability and optimal number of candidate endogenous reference genes under a range of experimental conditions (different N treatments and tissues) were analyzed using three software packages: geNorm, NormFinder, and Bestkeeper. Additionally, the stability rankings of selected reference genes were comprehensively evaluated using RefFinder [62].

The geNorm tool assessing gene expression stability (M-values) requires the conversion of raw Ct values to 2^−ΔCt^ values (ΔCt = original Ct − lowest Ct in each group). The geNorm algorithm also determined the optimal number of reference genes by evaluating Vn/n + 1 value, where the default threshold was 0.15. If Vn/n + 1 < 0.15, the optimal number of reference genes was n; otherwise, it was n + 1 [26].

The NormFinder algorithm employed an ANOVA-based model to assess the stability of reference gene expression by analyzing variations both within and between groups [36]. A lower stability value indicated a more stable reference gene, guiding the selection of the most appropriate internal control.

The BestKeeper tool calculated the expression stability of candidate reference genes by calculating the standard deviation (SD) and coefficient of variation (CV) from raw Ct values. Genes with lower SD and CV were considered more stable and suitable as reference genes [63].

RefFinder integrates the results from geNorm, NormFinder, and BestKeeper to rank the stability of all candidate reference genes, identifying the most consistently expressed gene [64].

### 4.6. Gene Expression Level Analysis Using Various Reference Genes

The sequences of *SsAMT1* (KAG5224131.1) and *SsNRT2* (KAG5238048.1) were identified through a BLAST search in the *Salix suchowensis* genome and selected as a target gene to validate the reliability of the reference genes. RT-qPCR was conducted to measure the expression levels of *SsAMT1* and *SsNRT2* under different N treatments for 24 h and across various tissues using specific primers (Table S1). Relative expression levels were normalized to the reference genes (*EF1α*, *EFβ*, *αTUB*) and calculated using the 2^−ΔΔCt^. Data were presented as mean ± standard deviation (SD) of four biological replicates. Significant differences were analyzed using one-way ANOVA followed by Tukey’s test in IBM SPSS Statistics version 21. Diagrams were created using Origin Pro 2025 (64-bit) SR1 10.2.0.196.

## Figures and Tables

**Table 1:** General information of the thirteen candidate reference genes in *Salix suchowensis*.

Gene	Locus	Protein Size (aa)	Gene Description	Gene Structure
*EF1α*	KAG5219160.1	229	elongation factor 1 alpha	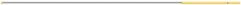
*EFβ*	KAG5253579.1	223	elongation factor beta	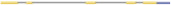
*αTUB*	KAG5254066.1	451	alpha−tubulin	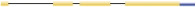
*βTUB*	KAG5249312.1	446	beta−tubulin	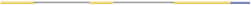
*GAPDH*	KAG5246615.1	453	glyceraldehyde−3−phosphate dehydrogenase	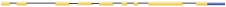
*18s*	KAG5248457.1	289	18S rRNA (guanine−N(7)) −methyltransferase	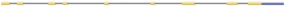
*Actin1*	KAG5252779.1	267	actin	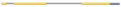
*Actin2*	KAG5255349.1	257	actin	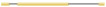
*H2A1*	KAG5232433.1	144	histone H2A	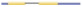
*H2A2*	KAG5246854.1	130	histone H2A	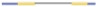
*UBQ1*	KAG5248096.1	135	ubiquitin	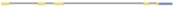
*UBQ3*	KAG5221099.1	186	ubiquitin	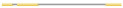
*H2B2*	KAG5239632.1	139	histone H2B	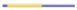
				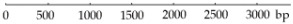 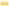 Exon 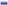 upstream/downstream − Intron

**Table 2:** Stability analysis of eight candidate reference genes using the geNorm calculation method. *EF1α*, *EFβ*, and *αTUB*, identified as the most stable reference genes, were marked in dusty pink, aqua, and grey-purple, respectively.

		Rank	1/2	3	4	5	6	7	8
	(Gene)_Stability_								
Treatments	NH_4_NO_3_		(*EF1α/EFβ*) _0.25_	(*αTUB*) _0.34_	(*βTUB*) _0.52_	(*H2A1*) _0.74_	(*Actin2*) _0.91_	(*Actin1*) _1.14_	(*GAPDH*) _1.62_
	NH_4_^+^		(*EF1α/EFβ*) _0.33_	(*αTUB*) _0.45_	(*Actin2*) _0.56_	(*βTUB*) _0.71_	(*Actin1*) _0.95_	(*H2A1*) _1.37_	(*GAPDH*) _1.70_
	NO_3_^−^		(*αTUB/βTUB*) _0.20_	(*EFβ*) _0.39_	(*EF1α*) _0.50_	(*Actin2*) _0.79_	(*Actin1*) _1.02_	(*H2A1*) _1.21_	(*GAPDH*) _1.73_
	−N		(*EF1α/EFβ*) _0.35_	(*αTUB*) _0.42_	(*βTUB*) _0.53_	(*Actin2*) _0.68_	(*H2A1*) _0.88_	(*Actin1*) _1.10_	(*GAPDH*) _1.50_
Tissues	Root		(*EF1α/βTUB*) _0.18_	(*EFβ*) _0.19_	(*αTUB*) _0.24_	(*ACT1*) _0.38_	(*ACT2*) _0.57_	(*H2A1*) _0.70_	(*GAPDH*) _0.83_
	Stem		(*EF1α/αTUB*) _0.29_	(*EFβ*) _0.32_	(*Actin1*) _0.35_	(*βTUB*) _0.39_	(*GAPDH*) _0.49_	(*ACT2*) _0.66_	(*H21A*) _0.83_
	Leaf		(*αTUB/GAPDH*) _0.23_	(*EF1α*) _0.26_	(*EFβ*) _0.28_	(*βTUB*) _0.33_	(*Actin1*) _0.38_	(*Actin2*) _0.44_	(*H2A1*) _0.87_
Total			(*EFβ/EF1α*) _0.35_	(*αTUB*) _0.44_	(*βTUB*) _0.59_	(*Actin2*) _0.80_	(*Actin1*) _1.04_	(*H2A1*) _1.26_	(*GAPDH*) _1.68_

**Table 3:** Stability analysis of eight candidate reference genes using the NormFinder calculation method. *EF1α*, *EFβ*, and *αTUB*, identified as the most stable reference genes, were marked in dusty pink, aqua, and grey-purple, respectively.

		Rank	1	2	3	4	5	6	7	8
	(Gene)_Stability_									
Treatments	NH_4_NO_3_		(*αTUB*) _0.123_	(*EFβ*) _0.176_	(*βTUB*) _0.268_	(*EF1α*) _0.348_	(*H2A1*) _0.544_	(*Actin2*) _0.620_	(*Actin1*) _1.321_	(*GAPDH*) _2.062_
	NH_4_^+^		(*EFβ*) _0.115_	(*αTUB*) _0.172_	(*Actin2*) _0.199_	(*EF1α*) _0.275_	(*βTUB*) _0.518_	(*Actin1*) _1.034_	(*H2A1*) _1.669_	(*GAPDH*) _1.739_
	NO_3_^−^		(*αTUB*) _0.069_	(*βTUB*) _0.069_	(*EFβ*) _0.145_	(*EF1α*) _0.432_	(*Actin2*) _0.560_	(*H2A1*) _0.955_	(*Actin1*) _1.273_	(*GAPDH*) _2.203_
	−N		(*EFβ*) _0.082_	(*αTUB*) _0.143_	(*βTUB*) _0.154_	(*EF1α*) _0.251_	(*Actin2*) _0.398_	(*H2A1*) _0.824_	(*Actin1*) _1.254_	(*GAPDH*) _1.804_
Tissues	Root		(*αTUB*) _0.173_	(*EFβ*) _0.210_	(*βTUB*) _0.217_	(*EF1α*) _0.231_	(*Actin1*) _0.488_	(*Actin2*) _0.502_	(*H2A1*) _0.602_	(*GAPDH*) _0.748_
	Stem		(*EF1α*) _0.192_	(*EFβ*) _0.234_	(*αTUB*) _0.316_	(*Actin1*) _0.316_	(*βTUB*) _0.335_	(*GAPDH*) _0.374_	(*Actin2*) _0.570_	(*H2A1*) _0.879_
	Leaf		(*αTUB*) _0.078_	(*GAPDH*) _0.078_	(*EFβ*) _0.174_	(*EF1α*) _0.241_	(*Actin1*) _0.254_	(*βTUB*) _0.292_	(*Actin2*) _0.371_	(*H2A1*) _1.463_
Total			(*EFβ*) _0.041_	(*αTUB*) _0.153_	(*EF1α*) _0.334_	(*βTUB*) _0.357_	(*Actin2*) _0.500_	(*H2A1*) _1.095_	(*Actin1*) _1.212_	(*GAPDH*) _1.951_

**Table 4:** Calculation of the SD and CV of the Ct values of eight candidate reference genes using the BestKeeper tool. *EF1α*, *EFβ*, and *αTUB*, identified as the most stable reference genes, were marked in dusty pink, aqua, and grey-purple, respectively.

		Rank	1	2	3	4	5	6	7	8
Treatments	NH_4_NO_3_	Gene	*EF1α*	*EFβ*	*αTUB*	*H2A1*	*βTUB*	*Actin2*	*Actin1*	*GAPDH*
		SD/CV	0.66/3.23	0.66/2.74	0.71/2.97	0.79/2.87	0.89/3.39	1.27/4.49	1.82/6.59	2.05/8.27
	NH_4_^+^	Gene	*Actin2*	*EFβ*	*EF1α*	*αTUB*	*βTUB*	*GAPDH*	*H2A1*	*Actin1*
		SD/CV	1.07/3.80	1.08/4.34	1.20/5.72	1.23/4.99	1.30/4.89	1.55/6.34	1.86/6.56	2.01/7.06
	NO_3_^−^	Gene	*EF1α*	*EFβ*	*H2A1*	*αTUB*	*βTUB*	*Actin2*	*Actin1*	*GAPDH*
		SD/CV	0.67/3.30	0.71/2.96	0.72/2.64	0.91/3.83	1.05/4.08	1.15/4.07	1.65/6.01	2.72/11.01
	−N	Gene	*EFβ*	*EF1α*	*αTUB*	*βTUB*	*Actin2*	*H2A1*	*GAPDH*	*Actin1*
		SD/CV	0.71/2.84	0.74/3.57	0.89/3.65	0.96/3.56	1.10/3.88	1.10/4.07	1.84/7.48	1.86/6.67
Tissues	Root	Gene	*αTUB*	*Actin1*	*EF1α*	*βTUB*	*EFβ*	*H2A1*	*Actin2*	*GAPDH*
		SD/CV	0.43/1.79	0.43/1.59	0.44/2.19	0.46/1.75	0.49/2.05	0.84/3.14	0.88/3.03	1.16/4.29
	Stem	Gene	*H2A1*	*GAPDH*	*Actin2*	*βTUB*	*EF1α*	*EFβ*	*αTUB*	*Actin1*
		SD/CV	0.73/2.66	0.87/3.48	0.94/3.46	0.94/3.75	0.96/4.65	0.96/3.95	1.05/4.47	1.11/4.24
	Leaf	Gene	*EF1α*	*αTUB*	*Actin1*	*EFβ*	*GAPDH*	*Actin2*	*βTUB*	*H2A1*
		SD/CV	0.48/2.25	0.63/2.55	0.64/2.10	0.69/2.75	0.70/3.22	0.77/2.68	0.78/2.86	1.64/5.86
Total		Gene	*EFβ*	*EF1α*	*αTUB*	*βTUB*	*Actin2*	*H2A1*	*Actin1*	*GAPDH*
		SD/CV	0.80/3.26	0.83/4.03	0.92/3.81	1.07/4.05	1.14/4.04	1.15/4.19	1.85/6.64	2.07/8.39

**Table 5:** Comprehensive ranking of the expression stability of the eight candidate reference genes based on the RefFinder tool. *EF1α*, *EFβ*, and *αTUB*, identified as the most stable reference genes, were marked in dusty pink, aqua, and grey-purple, respectively.

Rank			1	2	3	4	5	6	7	8
Treatments	NH_4_NO_3_	Gene	*αTUB*	*EFβ*	*βTUB*	*EF1α*	*H2A1*	*Actin2*	*Actin1*	*GAPDH*
	NH_4_^+^	Gene	*EFβ*	*αTUB*	*Actin2*	*EF1α*	*βTUB*	*Actin1*	*H2A1*	*GAPDH*
	NO_3_^−^	Gene	*αTUB*	*βTUB*	*EFβ*	*EF1α*	*Actin2*	*H2A1*	*Actin1*	*GAPDH*
	−N	Gene	*EFβ*	*αTUB*	*βTUB*	*EF1α*	*Actin2*	*H2A1*	*Actin1*	*GAPDH*
Tissues	Root	Gene	*αTUB*	*EFβ*	*βTUB*	*EF1α*	*Actin1*	*Actin2*	*H2A1*	*GAPDH*
	Stem	Gene	*EF1α*	*EFβ*	*αTUB*	*Actin1*	*βTUB*	*GAPDH*	*Actin2*	*H2A1*
	Leaf	Gene	*αTUB*	*GAPDH*	*EFβ*	*EF1α*	*Actin1*	*βTUB*	*Actin2*	*H2A1*
Total		Gene	*EFβ*	*αTUB*	*EF1α*	*βTUB*	*Actin2*	*H2A1*	*Actin1*	*GAPDH*

## Data Availability

Raw data will be made available upon request.

## Supplementary Materials

The following supporting information can be downloaded at https://www.mdpi.com/article/10.3390/plants14193101/s1: Figure S1. Analysis of thirteen candidate reference gene products using agarose gel electrophoresis (A) and RT-qPCR (B); Figure S2. Melting curve analysis of thirteen candidate reference genes in *Salix suchowensis*; Figure S3. The amplification efficiency of PCR primers of thirteen candidate reference genes in *Salix suchowensis*; Figure S4. Expression of *ammonium transporter 1* (*SsAMT1*) and *nitrate transporter 2* (*SsNRT2*) in roots, stems, and leaves of *Salix suchowensis* seedlings exposed to different N treatments for 24 h. Table S1. Primers used for RT-qPCR analysis. File S1. The sequences of the 13 candidate reference genes in *Salix suchowensis*.
