# Supplementary material for: EF1α and αTUB Are Stable Reference Gene Pairs for RT-qPCR-Based Gene Expression Studies in Salix suchowensis Under Nitrogen Treatment Conditions
Source: Plants (Basel). 2025 Oct 8;14(19):3101. doi: 10.3390/plants14193101 (PMC12526094; doi:10.3390/plants14193101)
Supplement: Supplementary file 1 [file plants-14-03101-s001.zip › File S1.pdf]

File S1. The sequences of the 13 candidate reference genes in *Salix suchowensis*.

Exon Intron Primer

**EF1a [protein_id=KAG5219160.1]**

ATGGAAGGGAAGGTTTGTTAAACCAGTGTGAACGCTTGCACGGAAAATACTACTTGAC
ATCTGTGTTTGGAAATGCAGTCATGTTTATTTTTTAAAATGTATTTTTTTTTATAATTATTT
TTAATATCGATATATAATAATAAAAATATTAAAAATTATTTTAATAACAAAAAAAAAATAC
TACAACTCCAATATAAGGTCGCATTGGTCATATAGCTTGTACGAGCCGATTCGCCCGCTC
CGCAGAGTTTTTTTTATCTATGTAAAAAATTGATTGATTTTATAAATATTGTATTTTGTTAT
TTTAATATTTCATGAGTAAAATATTAATTCATAAAATCTGTGAAAAATGGTTTCTTTCAAC
CAAAAAAGTGATTGAATATTACTATAGCTAAAAAACTTTTGATAAAATTTGTTAGAAAAT
TTGCTAGGAAGGTTAAATGATTTTTTAGAAATTTGTTGTAACGGATACTCTGGTTTAAAA
AAACACAAAAAAAGAAACCTCGTTTAAAAAAAAAAGATTTGGACACGACGGTTATTAA
AAAATGAAAAATGTGAAAAAAAAAGTCACAGTTTATTGATATCCATGATCAAAGTGGAA
AAAAAACAACTTATGTGATAATCATTCCTTATGTTATTAAAAAATGAAGTGCATGTGGA
ACACATTTGCTGGCTGCATCAATAAGCATGATTTATGTTTTTTCGTCAGAGTTTTTTATTT
ATGTAGAAAATTGATTGTTTTTATATGTATTGTATTTTGCTATTTTAATATTTAATTAGTAA
AATTATAATTTATGAAATATGTATAACTGGAATGGAAAGTGATTTGTATATTTTTTAATCAT
AGATAATGTAGGTAATTTTATTTTTAGAAGTTGCCTTTTTTCCCCACTAATATTAGATCAG
ACACATTATAAATAATATTTTTTTTTGCCGAAATAAACTTACATTTTTTGCATGCTATAATT
AGTTATTTCAATGTTTAATTACTAAAATTGCAATAGATAAAATATGTTAGAAATGATTTTT
TTTGTATAATTAGTATGATTGCCAGCACATTGCGCTGTGTTTTTTTCTATGCAGAAAACTT
ATTGATTTTTGACATGTTGTAAGTAGTTGTTTAAACATTTAATTAGTAACATTATAATATAT
CACAAATAATTTTTTCCCCGCCTAAAAGGAATAATGTAATTGAAATGGAAAGTGGTTTT
GATAATTTTTAATCGTAGATAATCTAGGTATATTTGTCAAAAGAAAAAAAAATTGAGTTG
TTTTTTCTCCAAAGTAATATTAGATTTGAAATATATATAAAGAAAATTTAAGAAATAAATT
TAAACAAAAGAGTATAAAATTTATATTTTTTTATCATTAACAAAAGAGCAACCAAGAAA
AAGAATTAAATTATTATTATAATTATAATTATTATTTTTTTTTATTTTTCTGCTAATAATGAA
AGAAAACAAAATGAAACACTAAAACAATTCTAAAAACCTCAACAAAAGAAAAAAAAT
ATCATTGTTATTACTGTACAAACAATAAAAATTATCAAATTATCCTGATAGAAAAAAATT
TAAAAACATTAACAAAAGAGTAAGCAAGAAAAAAAAAGTAGAATTATTATTATTATTATG
TTAATAATGGAAAAATAAATAAATGAAAGACTAAAAAGATCTTAAAAACTTTAACAAA
AGAAAAAAAAATTATCAAGTAAAAAAAAGTCATTTGATATAAAAATAAAAATAATAAA
ACGAATAAAAAACAAAAAGAAAGAACAAATAGGAAAAATAAAAACACAAATATTAAT
TTGCAAATGACCATAGATATAAAACAAAATTTAAGAACTAATTTTTTATGAAAAAATATC
ACCTTAACAAATAAGAAAATAACAAAAATTAAAAATCAAAATGAAGAGTACACTTATAT
ATACATTGACAAAAGTAGAGATTATTCTAGAAATAAAAAGAAATAAACGGATTAAAATA
CAAAAAAATAAAAGTATAAGGGTCGAACTGAAAATCAAATATACAGGGATTAAAATCA
AAAATTAAAAGTATAAGCTCCAAAATGAAAATTGCATATAAAATCAAATTTAATAACAG
GATGAAATTGAAGAAATCGCAAGTTCTGTACCGTATTTATTAAATAATTTCAAGGGCCC
AACTCTGCTGGAGGCCCTCGACCAGATTTCCGAGCCCAAGAGGCCCTCAGACAAGCC
CCTCCGTCTCCCGCTTCAGGACGTGTACAAGATTGGTGGTATCGGAACTGTCCCGGTG
GGACGTGTTGAAACTGGTGTCATCAAGCCTGGCATGGTATGCACTTTTGGGCCAACTG
GACTGACCACTGAAGTCAAGTCCGTTGAGATGCACCACGAAGCCCTCCTAGAGGCTCT
TCCTGGTGACAATGTCGGGTTCAATGTTAAGAATGTAGCTGTGAAGGATTTGAAGCGT
GGGTTTGTGGCCTCGAACTCCAAGGATGATCCGGCCAAGGAAGCCGCCAACTTCACCT
CCCAAGTTATCATCATGAACCATCCTGGACAGATTGGTAATGGTTATGCTCCAGTTCTTG
ACTGCCACACCTGTCACATTGCTGTGAAGTTTGCTGAGATCCTCACCAAGATTGACAG
GCGATCTGGTAAGGAACTGGAGAAGGAGCCCAAGTTCCTGAAGAACGGAGATGCTGG
TATGATTAAGATGCTTCCCACCAAGCCCATGGTGGTGGAGACTTTCTCATAGTATCCTCC
ACTTGGTCGTTTTGCTGTGAGGGACATGCGCCAGACGGTTGCTGTGGGAGTTATCAAG
AATGTGGAGAAGAAGGATCCCTCTGGAGCCAAGGTGACCAAATCTGCTGCCAAGAAG
GGTGGCAAGTGA

**EFβ [protein_id=KAG5253579.1]**

ATGACAGTCACTTTCTCAGATCTACACACAGAATCAGGCCTCAAATCCCTGAACGAGTA
TCTCTCCGGGAAATCTTATATCTCCGGAGATCAGATTTCCAAGGATGACATCAAAGTTT
ACGGTGCTGTTTTGGTGAATCCTGGTGGTGCTTTCCCAAATGTTGGCAAGTGGTATGAT
TCTGTTTCTTCACAGCTTGCCTCAAGGTAACAATTTTTCACTTCTTAAAGTTCAATTTCG
CATCTTGGGTTGATTTTGTTTTGATTTGGTTGGTGATTGATTATGGTTTATGTGTTTTGAT
TTATAGCTTCCCAGGAAAAGCCTGCGGTGTAAGAGTTGGTGCCGCTGCCTCTGCTCCG
GTTGAAGCTGCCCCTGCCAAGGAGGTTTCTGTCTTTCTTTGCAATAATTTTGCTTAATAT
CTGAATGATGATTTCTGGTTTTTGTCTTCAATTGGGAATCTCAAAGTCTGGATCTTTTTC
CATGCGTATGTAGTTTATACACTGTGTGATTTGATTAGCTTTCATTGCGTATAAAACCATT
GCCAATTGGGAGTAAAATAATGTAATCGATATAGAAGGTCAGATCTTATTACCCAGTAGC
GCACAGATAGGGCAGCTCTTACCTGCAAATGTATTCTCGCAAATTGAAGACTTCTGCCC
TGGAACCAGTGAGAGTATAGCTGCGCCTGTCCTATGAGTTTAAATTTAAAGCTTGACTT
ATGTTTCTCACTGGTGTGTTAGAGGTAGGATTCAATGATAATGCGTTATTTTAATGCAAA
AAATAGGTTTTTGTGCTGTTATGTTACATGTTGGGGATGACTATTCCGTGATCTCAGTGA
TAGGTCTAAGAGTAATTTGCTGGTGATTTACAAATCACGCTGAATTTGCTATTTGTACTG
TGCTTTAACTTTGAATTAGGAAAGTCCAGTACATTGGTTTTGTAGGTGCAAGTCACATG
GCAAAGATATTAGGGACGCTTCTTGACTTTCTTGCATTGAACTTGGAAGTTTTTCTTGT
TTTGTTTCAGGCTGCTGGTGATGACGACGATGACTTGGATCTTTTTGGCGATGAGACTG
AAGAGGACAAGAAGGCAGCAGAAGAGAGGGAGAAAGCTAAAAAGCAATCCGCCAAG
AAGAAAGAGAGTAAGCGTTCTCGGCCCACTTTCTACTTCACTCCAAAGTCTCGTTCAT
GCTCTTGTATAACATGTGTCAACTGTGACTAAAGTTCATCACTGCATTTGCCATCGACTT
CTTTGGCAAGAGTCAATTTTATTAAAATCTCAAGTTGCAATAAAGATAATATTTATTAATT
TATCTTTTTTTTTGAGTTAACATATAATTGGATAAAAAGGATAGATAAATAAATAGATATT
ATCTTTATCACAATAATGTTTATTTATTTTTAAAATATTATCTTTTAAAATTAAATAAATATT
ATTTTTATCTCAGAAAAAAGCCTCCTATTATGACTTTATTTTATATTCTGTTATGTTGATGT
TTCCCCACTTCTAGGTGGAAAATCTTCTGTTCTCCTGGACGTGAAGCCATGGGATGATG
AGACAGACATGGCGGCGTTGGAGAAGGCAGTTAGGAGTATTGAGATGCCCGGTCTTTT
ATGGGGAGCATGTATGAGTTATCTTGACCAAGTTTTATTTATTTATTGTTCTCACACTTGA
TTGTGCGATGCTAACCCTTGTGTGTTGTTTTTGTTAAGCAAAATTGGCTCCAGTTGGTTA
TGGAATCAAGAAGCTCCAGATCATGCTTACCATCGTCGACGACCTTGTATCAGTTGATT
CCCTCATCGAGGAGTATCTCACGGTCGAGCCCTGCAACGAATATATTCAGAGCTGTGAT
ATTGTTGCCTTCAACAAAATCTAA

**aTUB [protein_id=KAG5254066.1]**

ATGAGAGAGTGCATTTCGATCCACATTGGTCAGGCTGGTATTCAGGTCGGAAATGCCTG
CTGGGAACTCTACTGCCTCGAGCACGGTATTCAGGTATCTTTCGATAGATCTGTCAATTC
TGGTCTCATATCTTCCTATGTTCTTTAGATCTCATACTTTATACATGCGCACATTTACTTTT
GTGTTTAAATCTCGAGTTTTGTATGTGCTTTTGCTTGGATTTGTGGATTGTTTTTTGTTTG
TTTAGTTAGATCTAAACATTACCTGATGTGAAATTAATTTTTCGATTTTTGGGTTTGTTTT
GCTTGGTTTCCATTGGATCTGGGAATTTTTATTCTTATTTGGTTCATATTTGTTTATCATTG
CTTTCAAATTTGTTTAATATCGCCAGATCTTAGTGTGGCTCTTATCGTTTATCGATTTTGC
TTAGATCAACAAGTATATCTTTGCAAGCGAGTGCTTGCATCGTAAAGTCTTAACCTGGT
ATTGTCTTATTTTGGTGTGAAAGAATTTGTTGAATATTTTGTATATTCAACCGGGTATTGT
CAGAATATGAATATAATTTTTTCTCTTAATTTTATCATTATCGATATGAATTACTGATAAAT
GTGTGTGTATCATTTCTGTAGCCTGATGGCCAGATGCCAAGTGACAAGACTGTCGGTGG
TGGAGATGATGCCTTCAACACCTTTTTCAGTGAAACTGGTGCCGGGAAGCACGTCCCA
CGTGCCGTCTTTGTAGATCTTGAGCCCACTGTCATTGATGAAGTCAGAACCGGGACCTA
CCGCCAGCTTTTCCACCCTGAACAGCTCATCAGTGGCAAAGAAGATGCTGCCAACAAT
TTTGCCCGTGGACACTATACCAGTAAGTGTCCATTATTGTCTCTTCGTTTTCCTTTTTTGT
ATAGACTGGTGAGTTTATGATTCTAATGATTGTCTTTATTGTTTCAGTTGGCAAGGAAAT
TGTTGACCTGTGCTTAGACCGTATCAGAAAGCTTGCTGACAACTGCACTGGACTGCAA
GGTTTCCTTGTATTCAATGCTGTTGGCGGTGGCACTGGATCTGGTCTTGGATCCCTTCTC
TTGGAGCGTTTGTCAGTTGACTATGGAAAGAAATCCAAGTTGGGATTCACTGTCTATCC
ATCTCCTCAGGTCTCTACATCTGTTGTCGAGCCCTACAACAGTGTCCTCTCAACTCACT
CCCTGTTGGAACACACTGATGTGGCTGTGCTTCTTGACAATGAAGCCATCTACGATATC
TGCAAGCGCTCTCTTGACATTGAGCGACCCACCTATACCAACCTCAATAGACTTATCTC
TCAGGTATACATCTACTCCTTCCTGGGATGCTTTTGTTGATTTATTGATGCTCAGATAGAC
ATACAACATATGCTTTTGTATTCAATGCTTTTGTTGTTTTCATATAGGAAACTTCAATTCT
GTAGTTGTTGCAAATTTAAAGATGGTGCTGAACTGACCTCACTCTTTGCATGGAATTTG
CAGGTCATTTCCTCCCTGACCGCTTCTCTGAGGTTTGATGGTGCTTTGAATGTGGATGT
CACTGAATTCCAGACCAACTTGGTCCCCTACCCTAGAATCCACTTCATGCTTTCCTCCTA
TGCACCAGTCATCTCTGCTGAGAAAGCCTACCATGAACAACTCTCTGTTGCTGAGATC
ACCAACAGTGCCTTCGAACCTGCATCTATGATGGCAAAGTGTGATCCTCGCCATGGCA
AGTACATGGCCTGCTGCCTGATGTACCGTGGTGATGTTGTGCCTAAGGACGTCAACGCT
GCAGTTGCCACCATCAAGACCAAGCGTACAATTCAGTTTGTCGACTGGTGCCCCACCG
GATTCAAGTGTGGTATCAACTACCAGCCACCCACAGTTGTTCCTGGTGGTGATCTTGCC
AAGGTCCAGAGGGCTGTGTGCATGATCTCCAACTCCACCAGTGTTGCTGAGGTGTTCT
CTCGCATTGACCACAAATTTGACCTCATGTACTCCAAGCGCGCTTTCGTTCACTGGTAT
GTTGGTGAGGGCATGGAGGAGGGTGAGTTCTCCGAGGCGCGTGAGGATCTTGCTGCA
CTCGAGAAGGATTATGAAGAGGTAGGCGCGGAATCAGCCGAGGGTGAGGATGAAGAC
GGTGAGGAGTACATGTGA

**βTUB [protein_id=KAG5249312.1]**

ATGAGAGAAATCCTTCACATCCAAGCAGGCCAATGCGGCAACCAAATAGGAGCAAAG
TTTTGGGAAGTAGTATGTGCAGAACACGGGATTGACTCCACCGGTCGGTACAATGGTG
ACTCGGCTCTCCAACTCGAGCGAGTTAATGTTTACTATAATGAAGCCAGCTGTGGAAGA
TTTGTCCCTCGTGCTGTTCTAGTGGATCTTGAACCGGGTACTATGGACAGTCTTAGATC
CGGCCCGTACGGGCAGATTTTTAGACCGGATAATTTTGTGTTTGGCCAATCTGGTGCTG
GTAATAACTGGGCTAAAGGACATTATACGGAGGGCGCGGAGCTGATTGATTCTGTTCTT
GATGTTGTTAGGAAGGAGGCTGAGAACTGTGACTGCCTGCAAGGTAAGGGGTTTCGG
TTTTCATTTTTTGTGTGTTTTTGGTTTCTGTTTGTTTCTCGAAAAAGTGGAATGGTAAGC
GATACGGTTTTACTGTTTGTTTCTCAGGAAAGTGGACATGGAGTTCTTTTTTTCCTTTTC
TGTTTGGTTCTTCTGAAAATGATGAAAAGTTCAGTTATACGGTTTATATGGTCTGAGAA
ACTGAGGGAAATGGGATAAAGAGTTTCTGACTAGAGTTTTTCTTTTTACCTGGATTTTT
CCCCTTTAGTTTTCTGTTTGATTCTCTAGAAAGTGATTAGTGACGTTTTTTTGTTCGGTG
TCTAGTGAGGTCCTCTTGTCTGGTTTATGGGAAATGGAACCGAGTTTTTACTGTAGCTAT
TCCTGTCATTGTTTTTTTTCCCTGTTTGTTTCTGGGGAAAGTGGAGTAGCAGGGGAAAC
TAGGTATTTGTTTGGTTATCGGGAAAATGAAGGAATAGAGAGAGAAAATAGAATACATG
TGTTTTTGTACTAATGCAATAAAGTGAAGAAGCGAGTTGCTTTCATAAATTTGGCTAGT
CCACCTTCTACTCTAATCTCGTTGAAGGCAATTTGTTTCTGATAATGATTCAGCAAATAA
AAGAGAGTCTTGTCTGTGTTTTGGTTTTGGCAATTCGTTAAAAATAAATAAATGTTTTG
GTTTAAAAATGCAGGATTTCAGGTATGCCACTCACTGGGGGGTGGTACAGGGTCTGGA
ATGGGAACACTTTTGATCTCGAAAATAAGAGAGGAATACCCGGACCGGATGATGCTAA
CATTCTCTGTTTTCCCATCTCCAAAGGTCTCAGACACTGTGGTCGAGCCTTACAATGCA
ACTCTCTCTGTTCACCAGCTTGTTGAAAATGCTGATGAGTGTATGGTTCTTGATAATGA
GGCTCTTTATGATATCTGCTTCCGTACTCTCAAGCTCGCAACTCCCAGCTGTAAGCTCCC
CCCTTTCTGTTACCTCTTTTTTCCTGGTCTCAATTTTCCTTTCATTTGCTTCTCTTTGTGT
GTGTTTGTTGTGTACTTCGTTTGCAATTTTAGGAGCATTAGTTTAATTTATAATAGATGTG
CTGCCTGGATCAAAGGTTTCAGTTTCTTCCCTTTCATGTACCTGGACAATGAAGTAGGC
AAATAAAAGTTATCATAATCATGATTTATGTGGGACGGTAACATGTTAATTTGATCTGTG
TTTTATTTAAAAGTGGAGTTCGCCGTCTCATAATTGAAACAATATTCTAGTGTACCTAGA
AGAATATGAGAAAGTGAACAAAATATATCCTCGATTTTTCAGTCACCGCCTGTGGACGG
TTTTAGCCACATGGTTTCAGATTCTTTTCTGTCTCGATGCACAGATACAGCTGTGAGTG
CACGTGGGCCTCGCTTGCTAAAGCTGTTTTAGCTGATAAAGATATAGAACGTCAGGTTT
CGTAGGTTTTTGCCATTCTTAGTAGTGCTTTGGTAATAGCTTCAGCTAAACTAATACTTG
TTTTGATCTGCTCATTGTTATATCATGGTTGTCTATGGCTTGCTCCAAGTAAATAGATAAA
AAAAAAGGGACTTTGGGAACTTTCCTTCCCCTGTTATGTTATATAATTTTTCACTGTGAT
GAGATGATCAATGCGAAAGATGCCTTTATCCTAGATTGTAACTCACTGGAAAATTTTGT
TTATCAGTTGGGGATCTGAACCACCTGATTTCAGCCACCATGAGTGGTGTTACATGCTG
CCTTCGTTTCCCTGGTCAGCTCAATTCAGACCTTCGCAAACTTGCTGTGAACCTCATCC
CATTCCCCCGTCTTCACTTTTTCATGGTTGGCTTTGCACCTCTCACTTCCCGTGGCTCGC
AGCAGTACCGTTCCCTAACTGTACCTGAACTCACCCAACAAATGTGGGATTCCAAGAA
TATGATGTGTGCTGCTGATCCCCGCCATGGCAGATATCTCACAGCCTCTGCCATGTTTCG
TGGGAAAATGAGCACAAAGGAAGTTGATGAGCAGATGATCAACGTTCAAAACAAGAA
CTCATCCTACTTTGTCGAATGGATCCCCAACAATGTCAAGTCTACTGTCTGTGACATTCC
TCCTACAGGCTTGACAATGGCTTCCACTTTCATTGGCAACTCCACATCAATCCAAGAGA
TGTTCCGAAGAGTTAGCGAGCAGTTCACTGCCATGTTCCGCAGGAAGGCTTTCTTGCA
TTGGTACACGGGAGAGGGAATGGATGAGATGGAATTTACAGAGGCTGAGAGCAACAT
GAACGATTTGGTCTCAGAGTACCAACAATACCAGGACGCAACTGCCGACGAGGAAGG
CGAGTATGAAGATGAGGAAGAATACCAGGATGAGGCCTAA

GAPDH **[protein_id=KAG5246615.1]**

ATGGCTACCCACGCAGCTCTTGCCTCTTCAAGAATCCCTGCCAATACAAGACTTCCCTC
AAAGATCAACCACTCTTTCCCCACTCAATGCTCCTTAAAGGTTTCTCTTTCTCTCTATGC
TTTCCATACACAGTGCGTCCTAGCAAGTGCTCTTTTATGTGATGTTTTGAATATACTTGA
ATATGCAGAGGCTAGAAGTGGCTGAGTTTTCTGGGCTTCGAGCCAGTTCATGTGTAACC
TATGCCAAGAACGCTGGTGAGGGATCCTTCTTTGATGTGGTGGCTTCCCAACTTGCTCC
AAAGGTTTGCTAAACTATGCCTCCCTTCTTAACATTCCTTTCAATGATCATTAGAACATG
CCTCGTAATTATATTTTGCTTTAACAATATATAGTTCCACCTGAAAATTCTTCAGTGTAGT
TGTGTAATTGTGCATGATTATACATATTTGATAGCGTGATAGCTCGGGGAGGGAAATTTG
AACATGAAATCTCTCCTGTGAAGGTGACATGCTGATAACAAGGGGCTACAGCTTGACT
AGTTTGATTGCTTTATTTTAGCTGTAAACTTGGTATTTGGAAGTAGACCTTCATGTATCT
CCAGCGCACTGTATACTGGATTAATCTTGTACTGGATGTTTTCTCTAAAATTAGGTTGCA
GTTTCAACTCCTGTCAGGGCAGAAACTGTGGCCAAATTAAAGGTTGCTATCAACGGAT
TTGGACGCATTGGCAGGAACTTCCTGCGATGCTGGCATGGTCGCAAAGACTCTCCCCT
TGATGTAATTGTTGTCAATGACAGTGGTGGTGTCAAGAACGTAAGCAAGCAAATTACT
GTGTATATCTTAATTATCTAAATTTTGGAGAATTGAATTTCATTAATTTATTGATAAATCAA
CTAAGAATCTTATGCAACAAACAGGCTTCCCACTTGTTGAAATATGATTCAATGCTTGG
AACTTTCAAAGCAGAGGTGAAAATTGTGGACAACGAGACCATCAGTGTTGATGGCAA
GCCCATTAAGGTTGTTTCCAGCAGAGACCCTCTCAAGCTTCCTTGGGCTGAGCTCGGA
ATAGACATTGTTATTGAGGTAAGCTGTGCTCTCAATTCTGAAGTGAACTGTCAGTTTTG
TGGCAACTCTTTGTAAGATGATCATACCGCTGAACTGAATTGTGCAGGGAACCGGAGT
TTTTGTGGATGGTCCTGGTGCTGGGAAACATATTCAAGCTGGTGCCAAGAAAGTTATCA
TCACTGCTCCAGCCAAAGGTGCCGATATTCCAACCTATGTTGTTGGTGTTAACGAAAAG
GACTACGGCCATGAGGTTGCCGACATTATAAGGTTGGTATTTTAAACAATTCTGCATAC
AAAAAGCAAGTACCACTATTCTTGATCTTTCATTTCACTGCATATGATAAATTTTAAGCT
TATAAAGTTAAACTGTTTGCAGTAATGCTTCCTGCACCACAAATTGTCTGGCTCCCTTT
GTGAAAATCCTGGATGAAGAATTCGGTACGAATTCATTCACAATCAGTACTAAAATTTA
GATGCATATGAGGTAAACTTGAGTGATTGTGTGAGTTTTTTGGCAGGCATTGTCAAGGG
AACAATGACAACAACTCACTCCTACACTGGAGATCAGGTAACCATTAAGAGTTCAGTG
CTCTGCTGATTTACTGATATAACCTACTTAATTTGACATTGAAACTATGAAAGACTAATT
ACCACAACAAGAACAAATTGAATTATCGTTTGTGCTATCCAGAGGCTCTTGGATGCTTC
ACACCGAGACTTGAGGAGAGCCAGGGCTGCAGCATTGAACATAGTCCCAACAAGCAC
TGGTGCAGCCAAGGCTGTATCTCTTGTGCTGCCCCAGCTCAAGGGCAAGCTCAATGGC
ATCGCACTCCGTGTCCCGACACCCAATGTTTCAGTTGTTGACCTTGTTGTGAATGTTGC
GAAGAAGGGCATTACAGCAGAAGATGTCAATGGAGCCTTCAGAAAGGCCGCTGGGGG
GCCATTGAAGGGTGTATTGGACGTGTGTGATGTTCCTCTTGTGTCTGTTGACTTCCGAT
GCTCTGATGTTTCCTCAACCATTGACTCTTCATTGACCATGGTCATGGGAGATGATATGG
TCAAGGTTGTCGCCTGGTATGACAATGAATGGGGATACAGGTCAGAATCCTAAACTCAT
CATAACTCTCATTTGGCATGAATTAAATATTAAGCATACAATCCTGTATCCAGCTTGCCTA
GAACAGAATGTACATCAAAATTCGGATCCCTTATAAACTAGCAAGAGTACCCTAACTAT
AATGATATGATTTCGGTTTTCAGCCAAAGGGTCGTCGATTTAGCACATCTTGTAGCGGA
AAGTGGCCAGGAGTGGCTGCAGCAGGAAGTGGAGACCCATTGGAGGATTTCTGCAAG
ACAAACCCAGCTGATGAGGAATGCAAAGTTTATGAAGCTTAGATGA

**18s [protein_id=KAG5248457.1]**

ATGTCGAATCGGCCAGAGCTACTAGCTCCACCTGAGATTTTTATGATGATTCAGAGGC
TCGTAAATACACCTCCTCCTCTCGTATTATCGATATTCAGGTACTTTTTTTTTTTTTTTTA
TAAAAATAATAATTCTGTAATGCTTATTTAATGATTTTGTTAGCCGATGATGGTGTGTGGC
TGTTAACAGGCTAGGCTATCGGAAAGAGCATTGGAGCTTCTTGCTTTGCCTGTTGATGG
AATCCCTAGATTACTCCTCGATATCGGTATGTTGCATTTTAAATTTCCATCACAATCACAT
TATGAAGCCACTCTCTCTTCTCTGTTTGTAAATGTGATTAATTTATGGCTTATGGTTTGTT
TACCAGCTAGAAAATAGCTACTCCATTGTTTTCGTTATTTTTTTTTAGAATTATTATCTTGT
AGAGATCGAACATAGCGCCTTGTTTTAAACCTTGAATTTCTGATACTTGATACATTTGTT
TTTGTTGTTAATTATTCTGTCTGTGTGGAGAATTCTGTTCCAAAGTTTTTTGAATGCTTA
CTGTAATTGTTCCGGTTTTTTCAGGCTGTGGATCAGGGCTTAGTGGGGAGACATTAACT
GAAAACGGTCACCAATGGATCGGCTTAGATATTTCCCAGTCAATGCTTAGTATGTTTGC
TTGAACTTGTTCAATTTTGTAATATTTCACCGGAGGTTCATGTTAGGTTATTTTGCATTTG
TCTTTCTTGTCAAATCCAGATATTGCTTCGGAGAGGGAGGTTGAGGGTGATCTTTTACT
TGGTGATATGGGTCAGGTATCCAACTAAGATTCAGTTTTAGTTTATATACTTCGCAACAC
AATAAAAAAAAATAAAAGGAGAATCAAGGTTGTGGGGCAGAAATGCAATTTCTTGGCCA
GCATGATTTTTTTCTTTAAAAAAACTTATTTAGCAGGATGCCAGTTTGGTGCCTTTGTGG
CCTTATGTTGGGACCAGGTTGATGAGCTAAACATTAATCACTGATGCAAGGTTGTGCAA
TCTTCTAGTTTATTTCACTTTTGACCAAATATTCCCTGTCAAATGACACTTGAGGTTTAAT
ACTTAATGCTGTGTGCACTTATGATTTGTGAATGTTTGTACTTTTGGAAGCTATTCCAGA
TCAAGTCCTTTTTTCATTGTTATTACGATAGAATATGCAATTCAACCTGCAAAATAGGTT
TTTGATTCTTAATTATCGCTGAAATGCTTATATTATGTTGCAGGGCCTAGCACTTCGACCT
GGAATTATTGATGGTGCCATCAGTATTTCAGCTGTTCAGGTTTTACTCTCTTGCATGCTG
TAGACATTTATGCATGTTGAAAGTGTTTTGTGTTAGAACTTGTCCAAAAACTTCATTTTT
TTCCTTAATTCACGTCATTCGTTATTTTCAATGAACCTCTTTTATTTCACTCTGCTTCCTG
TAGTCTTCTTCTGATTTTACACCTATTTGAATTATTTCAGTTAAGTAGAGGCTTGCCTGTA
TTTTCCTTGTGCTCTTTGATGAACATGTCTTATATTGAAGGAAACAAAAATGTCTATTTA
TGAATTACTTTCCCCCCCATCTTGATTGTTCTCTCTTTCCACAACCTTCACTTGAAAAAC
AGAAGATTGCAGTTTTTTTAAAAAAGGCAAAAGGTTTCCCCCATCTTGATTGTTCTTCC
TAGCCTCGTATATTTCATTATTTTCTTTCATACCCTTTGTATGGGCTGTTTCTTAAATGTTA
AGTTGCGTAACACTTTAGTGAGTGTTTTTTTCTCACTTTCTTCTTTTAATATGGCTCCCA
GTGGTTATGCAATGCAGATAAGTCCTCCCATGAACCAAGACTTAGATTGAAGTAAGTTC
CTTCTCTTTTCTTTTGCGCTTTAGGGTTTTTGGGATTCAATTTTGAGCTTGGTTTCTTCTG
GATTTTTTTCAACTGGTTTTCATTAATGCGAAGCATTAAAGATTAATACGTCCATAACAG
ATTACTCAGAAGTAGGGAATTCACAAGAAACTCAAATATTCAATCTGACCAAACTCACT
AGTTTGAACAAGTTTTGTTGCATTCTATTAGATTTCTATGAACACTAGATAGTGATAAAA
AATAGCCATTTGAAGACTAGAGTCTTTGAATGACTGGTGCCTATTTTTCTCATTGTAAAT
TGCTTGACATTTTTATGGCAGGGCTTTCTTTGGATCATTATATAGATGTTTGGCAAGAGG
AGCAAGAGCAGTGTTTCAGGTGTATCCGGAAAACATAGCCCAGCGTGAATTAATTTTG
AGATGTGCAATGCATGCTGGATTTGCTGGTGGTGTTGTTATGGATTACCCACACAGGTA
TGCTAGGCTTCTATCTAGAAATCTCATGTTGAGTTTTTCATGACTGGTAAGCTCGAAGTA
TTATCTGTTGTACATTTTTTTTTCCGCTTGATTTTAATAGCACTGGCCCTACCCAACCCTA
TAACCTGAATGTTCTGGTTGGGAATAAAAACCTTCCCATAAAAGGTTGCTAGCTTTTCA
AGAATCTGAACCTTGAGAGCTAAATATGCTAGATGCTCCGTATGCTAATTCTCTGTTTTA
TCATGCAAGTATATTCACCCTGTTCTCTCTTTATCAATCAAGCTTACCTTCTGACATGTAA
TTTCTTGCTATTAATTATCCTCTGGTTTCATATTCCTCTTCAGGGTTTCATCATTGGCTGA
CTAAACTATTTTTCTGTCACAGTACCAAGAGTAGAAAAGAATACCTCGTGCTTACTTGC
GGACCACCGTCTATAAGCACTGCTGTTCCCAGGGGGAAAGGCGAAGACGGAGAAAGC
TGCTCTGAAGATGAAAGTAGTGAAGAAAACCAGATGGTGGGTTGCTTTGACTTGGAAT
TTTTGTTTCTTTTCAAGCTTTAACCGTGCTGTTTGTAAGCATGGTATCCTTTTCTTTTCAA
TTCAGATTTCTAGAATCACTAATAAACATGTATCCTGAGAATGCAGGTTTGCATTTCAGA
CAGGAACAGACCTAAGAAAAAACAGAAAATTTGCAGGAAAGGGAAGGGCAGAGAGT
GGATATTTAAAAAGAAAGAGCAACTGAGACGAAAGGGAAACGCAGTGCCTCCTGACA
CAAGATACACGGGTCGAAAACGGAAGGCTCGGTTTTGA

Actin1 **[protein_id=KAG5252779.1]**

ATGGCAGAGAGTGAGGATATTCAGCCTCTTGTTTGCGACAATGGTACCGGAATGGTCA
AGGTATTTAGCTAGATTTGTCGGGGAATTTGTGATCCACTTTTGATTCTTACAGTGATAT
TGGTTGTGATTGCTGATTTGGATTGGAAATTTCAGGCCGGGTTTGCTGGGGATGATGCA
CCAAGGGCTGTTTTTCCAAGCATTGTGGGTCGCCCACGCCACACTGGAGTGATGGTTG
GTATGGGTCAAAAAGATGCCTATGTTGGCGATGAGGCTCAATCAAAGAGAGGTATTTTG
ACTTTGAAATACCCAATTGAGCATGGTATCGTTAGCAACTGGGATGATATGGAGAAGAT
TTGGCATCACACCTTCTACAATGAGCTCCGTGTGGCTCCTGAAGAACATCCAGTTCTCC
TTACAGAAGCTCCTCTTAACCCCAAGGCCAATCGTGAGAAAATGACCCAGATCATGTT
TGAGACCTTCAACACTCCTGCAATGTATGTCGCTATCCAGGCAGTCCTTTCCCTTTATGC
CAGTGGTCGTACAACCGGTGAGTGATATGGTCCATCCTCTATTTGCTTTTATGTTCAAAA
TGATGAATGGAAAAAAAATTGTTATTGGCTTGCTGCATTGGAAATTTTATGAATGAAGC
GGAATTTTAGTGATTGGTTGGAAAAAAATAAATTTCAGGAAAGAAATTAAATTTATCTTG
ATCAAGATAAAAAAATGGAATGTCTTGGATAACTTCTCAAACCTCATTGTGTCTAATTT
GTAAGTTAACACGATCTATTTTTCACCCTCGGCCATAATCACATTTGCTTTTGGAGAATT
TTATATGATATATACAGGGATCATGATCATGAAAACCAAAGAACTGTCTTTGACAGCATG
TAGGTAACCGGCTTGGTGATTTACATTAGGGATCTTTTAATTGTTGAGATAAATTAGGAG
AAATAACATTTTTCATGATATATGTGTTTTCGCTAGGTATTGTTCTGGACTCCGGAGATG
GTGTGAGTCACACAGTTCCCATCTATGAAGGCTATGCCCTTCCACATGCCATTCTGCGT
CTTGACCTGGCAGGCCGTGATCTCACTGATGCCCTCATGAAAATCTTGACTGAGCGTG
GCTACTCTTTCACAACCACAGCAGAGCGTGAAATCGTAAGGGACATGAAGGAAAAAC
TAGCCTACATTGCTCTTGATTATGAGCAAGAGCTAGAGACAGCAAAGACCAGCTCATCT
GTTGAGAAGAGCTATGAATGCCAGATGGGCAGGTTATCACTATTGGAGCTGAACGTTTC
CGCTGTCCTGAAGTCCTCTTCCAACCGTCCATGA

Actin2    **[protein_id=KAG5255349.1]**

ATGACGCAGATTATGTTTGAAACATTTAATGTCCCTGCCATGTATGTTGCCATCCAAGCA
GTGCTCTCCCTTTATGCTAGTGGACGTACTACAGGTGTGTATGGGATGCGTATATATGTG
AGAGCTCTTCTCTGCTTTCAATCGTTCAGAAAAAAATTGGTGCTTTTCAAAGTTGACTG
TAACTTTCTTTTTCAGGTATCGTGCTGGATTCTGGTGATGGAGTTAGTCACACAGTTCCC
ATCTATGAAGGCTATGCACTTCCCCATGCAATCCTTAGGCTTGATCTTGCTGGAAGGGAT
TTAACAGATGCCTTGATGAAGATCCTTACGGAGAGAGGTTACACATTCACCACAACAG
CTGAACGGGAAATTGTAAGAGACATTAAAGAGAAGCTTGCATATGTGGCACTTGATTAT
GAGCTGGAGCTTGAAACAGCTAAAAGTAGCTCTTCAATTGAGAAGAACTATGAGCTAC
CTGATGGCCAGGTTATCACCATTGGTGCAGAGCGCTTCCGTTGCCCTGAGGTCCTATTT
CAACCATCACTCATTGGAATGGAAGCACCTGGAATTCATGAAACCACTTATAATTCGAT
CATGAAGTGCGATGTTGATATCAGGAAAGACCTGTATGGTAACATAGTGCTCAGTGGTG
GATCTACCATGTTTCCAGGAATAGCTGATCGAATGAGCAAGGAGATCACAGCTCTTGCT
CCAAGTAGCATGAAAATCAAGGTGGTTGCTCCTCCTGAGAGAAAATACAGTGTTTGGA
TAGGAGGGTCTATACTGGCTTCCCTCAGCACTTTCCAACAGGTATGTATTGCACACCTC
CATTTTCTCCGTTTAATTGTTGTATTTGATGTTGCATGTGGTTAAGTCATTCTTGACCCAA
AAGCTCAAAGGAGTTTAAAGGTCACCAGAATCACATACTTGCAAAAGAGAGGCTAAA
AGGTGAAGGAATGGAACAGAACAAATAATAACTTCCATTAGTTGAGAAACACATGCAT
GCATGCAGGAGAACAACACTCACAAGACAATTGGTAATGGTAGTTGGTACAAAATAAA
ACAACCAAATTAGAAAACCAAGTCATAACATATATTTATTTGGCTTTCGGCATCTTTATC
CAGGCAATTGATTTTAGATTTTAATTTTCGATTTGCTTGGTTTCTGTTCCATGCAGATGT
GGATATCAAAGGCAGAGTATGATGAATCAGGACCCTCCATTGTTCATAGGAAGTGCTTC
TAG

H2A1 **[protein_id=KAG5232433.1]**

CTCTCAACTTCCATTTCCCTCTAAGAAAACGCAATCGCCTTCAACAAATCTGAAGGTCT
TTTCGTTATTGAATCTGTGCAACAATGGAGGCAACAAAGACAACGAAAGGCGCCGGA
GGAAGGAGAGGAGGAGAACGAAAGAAGTCAGTTTCGAAGTCAACAAAAGCTGGTCT
TCAGTTCCCAGTGGGTCGAATCGCCAGGTTCTTGAAGAAAGGTCGTTACGCTCAACGT
GTTGGTTCTGGTGCTCCTATTTACATGGCTGCTGTTCTTGAATATCTTGCTGCTGAGGTA
TAACAGAAGTTGTCTTTTGCATCTGGTTTATTTTAAAAAGAAAAATGTCTTATAGCCTGC
AGCGTTGAAATATCTCGAGATCTGATTTTTTCATTGTTAATCCTATTGTTCTTTAAAGTTT
GTATCTGAATTTAGCCCTGATCTGATTTATATCTGTGTGTGATAAGGTGCTGGAATTGGC
CGGAAACGCAGCAAGAGACAACAAGAAGAACAGAATAAACCCAAGACACGTGTTGC
TGGCTGTAAGAAACGATGAAGAACTGGGAAAATTACTGCACGGCGTTACAATCGCAAA
CGGAGGAGTGTTACCAAACATTAACCCAGTTCTTCTACCTAAGAAAAGTGCAAGCAGC
GAGAAATCGTCTGGATCTGAGTCCAAATCTCCTAAAAAGGCCTAAGTCAATGGTTCTTA
GTTTTATTTTGTGGTAAAAAAATGTCTAGGATGTATCCTCCAATGTAGGAGGAACTTAGA
TAGGCAGTTTAGATTTTGTTTTGTAGGAAATAGTTGTCATATCTTTTGCACAAAAGAAG
AAATTTGTAGCTCTCACATGTTCATCAATCAAAAATGGAAAGCAAGCTTCTTGTATTGC
TAGCTTTCTCCTGATGGGATTTGAGTTTGATATTCATAGCTCGCTCGTGAGAATTTTTCA
AGAGTATTTATTTG

H2A2 **[protein_id=KAG5246854.1]**

ATGGCAGGAAGAGGAAAGGCTTTGGGGTCTGGAGTAGCAAAGAAAGCAACCTCAAG
AAGTAGCAAGGCGGGATTGCAGTTCCCTGTGGGGCGTATTGCTCGGTTCCTGAAGGCT
GGGAAATACGCCGAGCGTGTTGGTGCTGGTGCTCCTGTCTACCTCGCTGCTGTTCTTGA
GTATCTTGCTGCTGAGGTATAAAGCTACCCTTTTGGTGTTTTTTTTTTTTTGTGTTTAGTG
CGCACTGAGAAAATTTTCTGTATTTAGGAATGTGAATATTTTATGTTTTTGTCGGTTTTT
GAGAAGTTTGGGTACATGGGTATTCATTGTTTCTCAAGTTTTAATTTTTGGTTAATGGGT
ACATGGGTTCTAGTGAATTTAAAAAAAAAAAAAACCGTGCCTAGCAGTTGATTTTCACT
AGTTACAGGGTGCTTTGTTACTAAATATTTCGGGAAAATCAAAAGGAAACTTGGTTTAA
GCTTTCGGCTCTGGTTATTAAATTGGAATTCAAATCTTCTGGGTGTTTCTGTATTTTTTTT
TCATGATTCTTTATCCGGACTTGACATTAGCTCAACATCAGGAACGTTTCTTTTATTTGC
TTCAATTACATTTGGTGTTTTGTTGTTGATGTGTAGGTGTTGGAACTGGCTGGGAATGC
AGCAAGGGACAACAAGAAGACTAGAATAGTCCCAAGGCACGTTCAATTGGCAGTGAG
GAACGATGAGGAGCTGAGCAGGCTACTTGGCCAAGTCACAATTGCTAATGGTGGTGTC
TTGCCTAATATTCACAGCACTTTGTTGCCAAAAAGGGTTTCTAAAGGCCCGGTTGATGA
TGAATGA

UBQ1 **[protein_id=KAG5248096.1]**

ATGCAGATATTTGTGAAAACCCTAACTGGGAAGACTATTACTCTCGAGGTTGAGAGTGA
TGACACAGTTGACAACGTCAAGACCAAAATCCAAGACAGGGAAGGTAATCCTATTTTG
CTGTTCCTTTTTCATATCATATGCTTTTGTTATTTTAGGGTGTTTGGCGTTGTGTTTTAAA
AGTATTTTTTAAAAAAAAATAATATTTTTATTTACACTTTAAATTAAAAAAAAAATAATGTTT
TTAGATTATTTTCATGCGGTGATATCAAAAATAATTTTTAAAAAATAAATAAAAAAACATC
ATTTTGATACATTTATGAATAAAAAAACATTTTAAAAAATAACCGCAACTACAATCGCA
AACAGAAACTAAGTTTGCCAAAAACAAACAAACTGTGTGAGCATGTAGATGGTCTAA
GCTTGCATGGTAATTGTTAATAGGGATTCCGCCGGAGCAACAGAGGTTGGTTTCGCCG
GAAAACAGCTTGAAGATAGCCGTACCTTAGCCGATTACAATATCCAGAAAGGCATGTTA
AAAAAACTAGAAGGGCTTCTGTTTTTTTGCAGTGAAATATAGATTTTTTGAAATGTCTTT
ATTGATTTATCGTGGCTGGTGGTGTTGTTTAATTAGAATCCACACTGCATCTAGTCTTGA
GGCTTCGAGGAGGAAAGGGAGGTCCTAGCATGAAAATCGAGCCCTCCCTGAGGGAAC
TAGCTCGTAAATTCAATCAATACAAGATGATTTGCCGCAGGTACGTACACCTTTTCTCTT
ATCCTCGATTTTCAAACCCTAGAACTGATTAATGAACTCTTAATCAGCGGGGCATGCATT
AATCGACAAGCTCTTGATTTAATCAGAAAGTAACATTATGGATCGTCATGTAGATTTTGT
TTCATAGCTGCAGCCAGTTTTGATGAGATGCATCACAAAAGTATAATCATCCCTTTTAAG
ATCACATTATCAAACTAAACTCTCCGGCTTAATTAAGGATCAAGAAAGTATTCCAACAT
GAACAGGCTTTTGATCCACCAAACCTGTAATATGCCAAGATAATCAATTTTGTTTCGGC
TGTTGCTTTATTTTTTCAATCGAAATGGAATATTCTGGTTTCGAAGTGTTTTGACATGCT
GTTTTGGGTTATACTGTTTGTGTGTGTGTGTGTAAATATATTGATTCAAAAAAAGTTTAG
TGCAAATCCAAAATTATTTAAAATTATGCAATTAATCTAAAAAATACACGCTAAAATGAT
TTTTCTGAACAAAAACATGAAAATATATAATATTACGAGAATATCATCATCAATCTTGCA
AGGTTATTACATTATCTGATTGCTCTGAAATTTTAACATAATATATATAAATATATCAAGAG
ACTCCTCGTAAGAGTTCATGTCAATCTAACAGCTGGATTAAAAGATATGATTGATATTGT
AAAATTATTTATTATCTAAAATTAAAAAAAAAATATTATTTGGCTGAAACAAAACGGGC
GAAACGAAACCGAAACGTTTTGACCAAAATTTAAACCAAGATAAAATCAGCATCGTTT
TATCTCGTTTTTTTAACTGGTATAAAACATTTCAATCAGATCGGCCAAAACCAAACAAA
ACAGACAGACTTGATATATACACATTGCATTGAATTGATGTGGAAATAAACAGCAGAAT
ACTAATTGTTTTTTCAAGCCACTGTATTGCCTTGCTAGATGCTATTGTCGTCTGCCTCCC
AAAGCTAAGAACTGCAGGAAGAAGAAGTGCGGCCATGGCAACGAGGTTAAGTGTTAT
TATATATTTTCTTGATATGATTTCTCGGTGCTGTCAGGACATGCTCTGATACCTTCTATTTT
CTACTGGCAGTTGAGGCCGAAGAAGATGCCCCAGGGCTAG

UBQ3    **[protein_id=KAG5221099.1]**

ATGGCGGGTGCGGCACCAGACACGCCAATTAGATCACTGCCTTCATGGGCTCACCAA
GAAGCTAACCAGATACCAGAGGAGGCCATTTCTGCTCTAGAGACGTATAAGAAGAAG
GATCCAGCCAAAGGTGACCCCTGCTAGTAGTCCTCCCTCATTGCTAGCTGACATTACC
ATCTGAACAGTCGTCGTACGCTTCAAAGCTGTTGGAAATGCGCCAATTATGAAGCAG
AACTTCTACAAGATCACCGCGTCGAACAGGTTTCAGGCTGTGATTCAGTTCTTGCGAA
AGGAATTGGGTTGGAAGACAGGAGACCCACTGGTCCGTTGCATTCACAGCGAATACG
GTTTTGCGTGTTCATTCGGATCCCAGTTCACGTACATCAACCTGGCATTCTCGCCTGCC
CCTGATGACACGGTGTCGAACCTATTCAAGGTTTTTATCACTATTTGAACACGAAGAT
TCTGCTGATGATATACCTCCCCAGTCGTTTGCTACGGATGGACACTTGATTGTTAACT
ACAGGTATTTTCTGTCCTGAATTTTCGCTTCCGATTTATCAAATTGATGTCTTTCCCTA
GTACAACAGCAGCGTGGGGCTGATAGCAATATATCAATAAAGTATCTCTGCTATACA
CATGATATAGACTAGTTCTCAACACGCGATGATGCTTCAAGTGACCGACAGTACAGG
TGTTTGGCCTGCAAGTGGCTTCGATCCTTGTTCTTTGATTTGGCCATAGCTCAAACGGC
TTCCTGTGACTAGCCCGAGTGTCGGACGGTGGAACGACCGTTCTGCTGTCTGATGCGT
TTCTGCCACTAGCTATGTATATCTCTCTGTCGTTAACTTGCCGTACTCTTTTTGGCTCA
CTCCTCGTTATAATTCGGCGGCGTTTTCTCTGAAGCAAACTAGACAGAACCTAATAAA
TGTGTAACAATTGATATGTATGTTAAAGCGACGTGTCCAAACCACGGCTTCGGTGGA
GATTGGTAGGAGATCACTGGCTATCACCGTCCTTAGTCAGTCTTTGAACTCCTAGGCT
TATCACATTATCGTGATCCTCCCCAGAGCAAGTGCGGTGGCCTGGCCTTTGGGGGCAC
ATCCTCGAGAAGAAACTCCCCCGCCCCTACCTCGGGAGCTCATTATCCTATTCGTTTG
TAATGTGGAATAGAGAATATACGACTTCGCTCAAAATTTTCATCATGATATAGGTGCT
TTACCTGTCGTATTGATGTATCAGGCTAACCTTCGAGAGTCGCGGGGCCTTCTCCATA
ATCTGGGTGCCCGGATGTTTGCACCGATACGAATACAAGACTCACGCGTTGATCTTGC
TGTCCACGGCCAACCAAATAGCGTCCGGAATGTCCCTCAAAGTATATTTGACAAGCT
GAAGACCGAGATGAGGGTTAAGATCAAGACCTGA

H2B2 **[protein_id=KAG5239632.1]**

GAAAGCATTCTCCTTTTCCCTACTTAATTAGCCTCTCTCTTTCGAGTTTCAAGAATCCCA
AATGGCACCCAAGGCCGAGAAGAAGCCGGCCGAGAAGAAGCCAGCAGCAGCTGAGA
AAGCACCAGCCGAGAAGAAGCCAAGAGCCGAGAAGAAATTGCCCAAAGAAGGAGCT
ACTGAAAAGAAGAAGAAGAAGGCAAAGAAGAATGTGGAGACTTACAAGATCTACATC
TTCAAGGTATTGAAACAAGTCCACCCTGATATTGGGATCTCAAGCAAGGCTATGGGTAT
CATGAACAGTTTCATCAATGATATCTTTGAGAAGCTTGCTCAAGAGTCTTCAAGGCTTG
CCAGGTATAACAAGAAGCCTACTATCACGTCTCGGGAGATCCAGACTGCTGTCAGATT
GGTTTTGCCTGGAGAACTTGCCAAGCATGCAGTCTCTGAAGGGACTAAGGCTGTTACG
AAGTTTACTAGCTCTTAGGTTATTCATTTTTGTCGAGCTTTTGTGGGTTCTGCGGAATTA
GGGTTTATGTTTGATGTAAAGGTTATTTAGGTCCTGATCTGAACAAAATCCATGCACTTT
CTTTTGTTCTAATCAATGAAACTTTTTCGTAGCTCCTAAATGGTCTTCATACCTGTATTCA
ATGATTTGGTTCCGCGTCCCATATCTGTGCCTTGATTTTCTGGGAGTAGTTAGGGTTGGA
GGATTTGGGGGTTTTCCTTGACTGGGTCATTTGTGGCCAACAGGGCTGCAGGCCTACG
ACTTTTCCGTAAAAACAAACCCTGTTTTGCAAATCTCTTTCTATCCTCCGCACCAAGAG
GTTGGCCCCGTCTTGTGGGTTTTCAAAATTTGTGATCCAACTGCAAAACGAACGTTTTC
TTTTAGATTGGTGTAATTTTTTCATTCACCAGGGAATCAGTCTTAAATTGTAT
