# Supplementary material for: EF1α and αTUB Are Stable Reference Gene Pairs for RT-qPCR-Based Gene Expression Studies in Salix suchowensis Under Nitrogen Treatment Conditions
Source: Plants (Basel). 2025 Oct 8;14(19):3101. doi: 10.3390/plants14193101 (PMC12526094; doi:10.3390/plants14193101)
Supplement: Supplementary file 1 [file plants-14-03101-s001.zip › Supplementary Materials.pdf]

Supplementary Materials

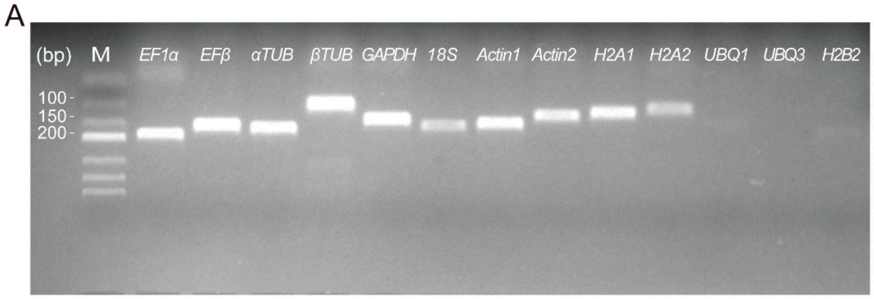

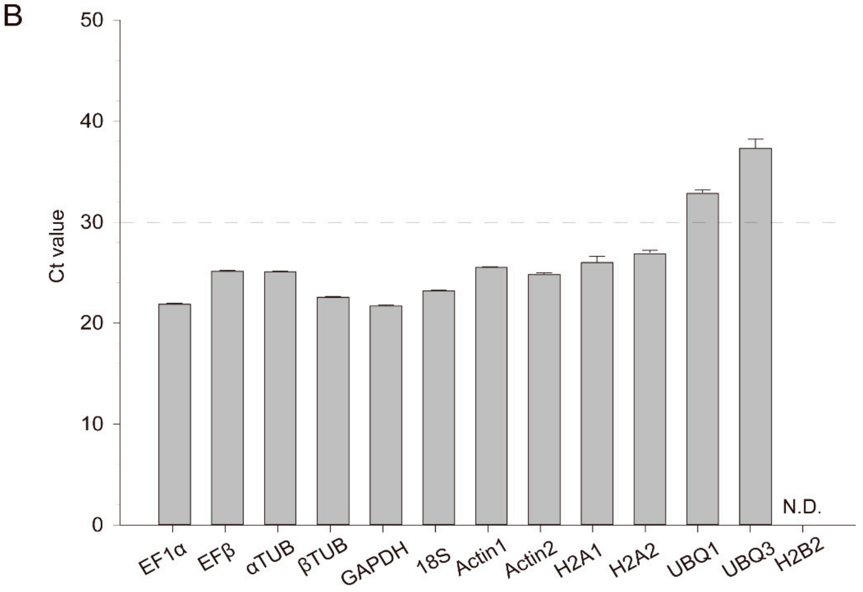

Figure S1. Analysis of thirteen candidate reference gene products using agarose gel electrophoresis (A) and RT-qPCR (B). M, molecular marker. N.D., not detected.

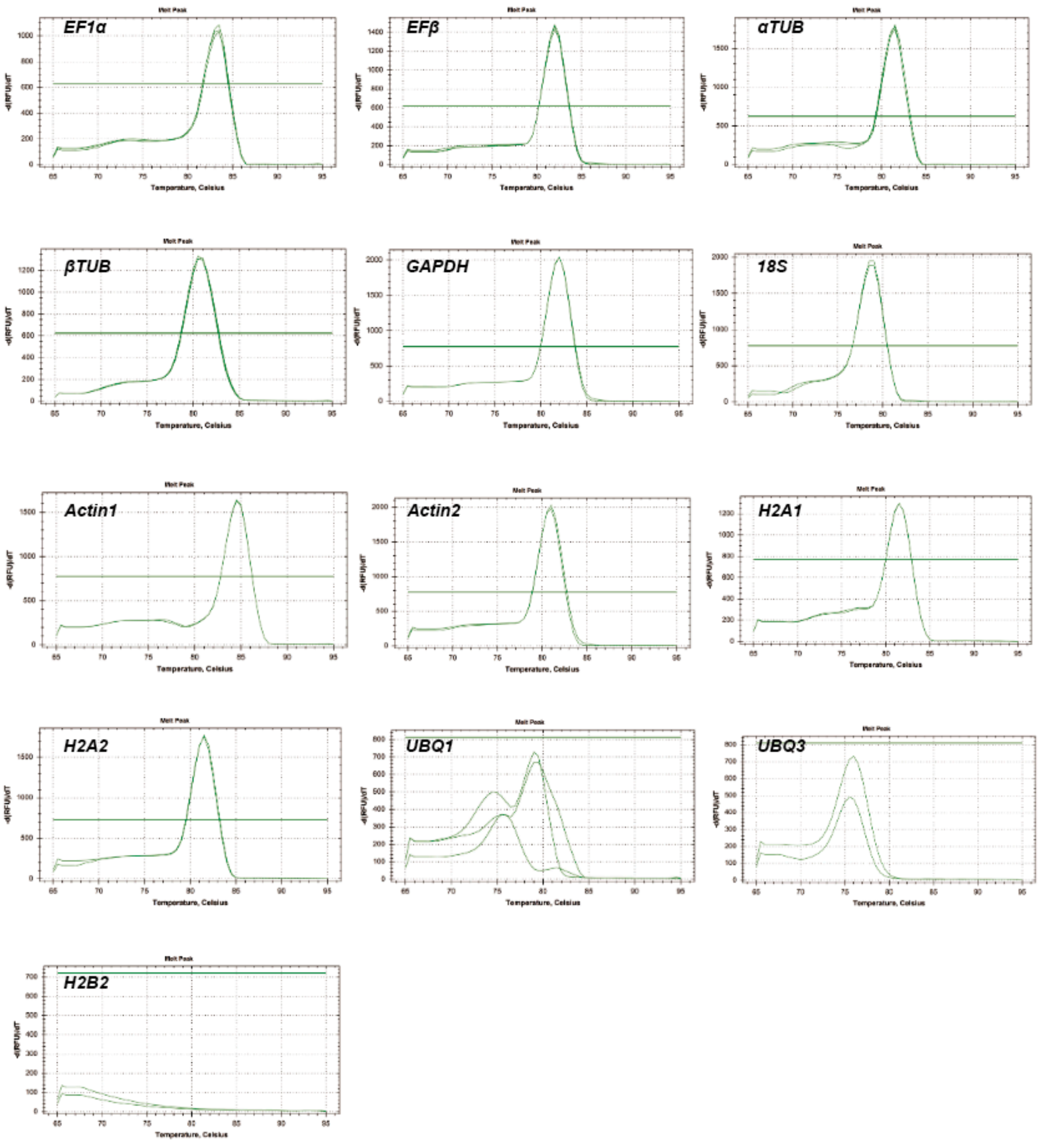

Figure S2. Melting curve analysis of thirteen candidate reference genes in *Salix suchowensis*.

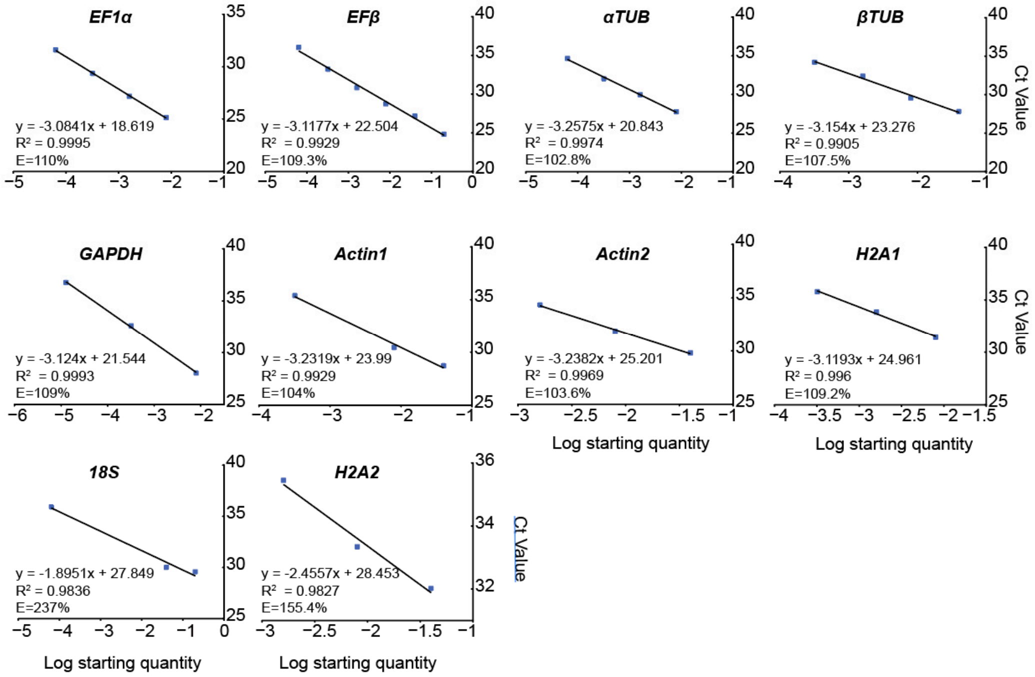

Figure S3. The amplification efficiency of PCR primers of thirteen candidate reference genes in *Salix suchowensis*.

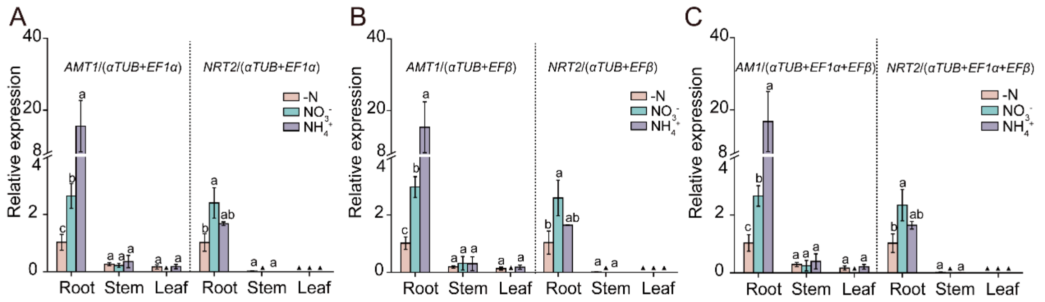

Figure S4. Expression of *ammonium transporter 1* (*SsAMT1*) and *nitrate transporter 2* (*SsNRT2*) in roots, stems, and leaves of *Salix suchowensis* seedlings exposed to different N treatments for 24 h. Relative expression levels were normalized to *αTUB* + *EF1α* (A), *αTUB* + *EFβ* (B), and *αTUB* + *EF1α* + *EFβ* (C), and calculated using the $2^{-\Delta\Delta Ct}$ method, with gene expression in roots under -N treatment serving as the control. Different letters indicate significant differences at $P < 0.05$ of the same gene among different N treatments. Values are means ± SD (n = 3-4 biological replicates). (▲) Value lower than 0.001.

Table S1. Primers used for RT-qPCR analysis.

Primer name	Primer Sequence (5′-3′)	Product size (bp)
EF1α	TGAAGAACGGAGATGCTGGT<br>TTGGCAGCAGATTTGGTCAC	172
EFβ	GGCGATGAGACTGAAGAGGA<br>CTAACTGCCTTCTCCAACGC	158
αTUB	GAGCGACCCACCTATACCAA<br>TGGTGCATAGGAGGAAAGCA	165
βTUB	AGCAGTTCACTGCCATGTTC<br>ATGTTGCTCTCAGCCTCTGT	97
GAPDH	ACCCTCTCAAGCTTCCTTGG<br>CTTTGGCTGGAGCAGTGATG	138
18s	CGGAGAAAGCTGCTCTGAAG<br>TCCCTTTCGTCTCAGTTGCT	154
Actin1	CGACAATGGTACCGGAATGG<br>TGAGCCTCATCGCCAACATA	147
Actin2	GCAGTGCTCTCCCTTTATGC<br>AAGCCTAAGGATTGCATGGG	123
H2A1	GGAGGAAGGAGAGGAGGAGA<br>CGTTGAGCGTAACGACCTTT	116
H2A2	TACCTCGCTGCTGTTCTTGA<br>TGAACGTGCCTTGGGACTAT	104
UBQ1	GCCGTACCTTAGCCGATTAC<br>TGCTAGGACCTCCCTTTCCT	87
UBQ3	GAGGAGGCCATTTCTGCTCT<br>CAGCCTGAAACCTGTTCGAC	139
H2B2	TCTCAAGCAAGGCTATGGGT<br>CATGCTTGGCAAGTTCTCCA	168
SsAMT1	CTGTCACCACAACATTGGCA<br>GCAACGAAGCCACAGATGAT	181
SsNRT2	AAAGAAGAAGGGCGATGCTG<br>AATGAGACCAGCTGTGTGGA	190
